# Photoactivatable Surface-Functionalized Diatom Microalgae for Colorectal Cancer Targeted Delivery and Enhanced Cytotoxicity of Anticancer Complexes

**DOI:** 10.3390/pharmaceutics12050480

**Published:** 2020-05-25

**Authors:** Joachim Delasoie, Philippe Schiel, Sandra Vojnovic, Jasmina Nikodinovic-Runic, Fabio Zobi

**Affiliations:** 1 Department of Chemistry, Fribourg University, Chemin du Musée 9, 1700 Fribourg, Switzerland; joachim.delasoie@unifr.ch (J.D.); philippe.schiel@gmail.com (P.S.); 2 Institute of Molecular Genetics and Genetic Engineering, University of Belgrade, Vojvode Stepe 444a, 11042 Belgrade, Serbia; sandravojnovic@imgge.bg.ac.rs (S.V.); jasmina.nikodinovic@imgge.bg.ac.rs (J.N.-R.)

**Keywords:** diatoms, drug delivery system, HCT-116, porphyrin, vitamin B_12_, carbon monoxide releasing molecule, rhenium, cancer

## Abstract

Systemic toxicity and severe side effects are commonly associated with anticancer chemotherapies. New strategies based on enhanced drug selectivity and targeted delivery to cancer cells while leaving healthy tissue undamaged can reduce the global patient burden. Herein, we report the design, synthesis and characterization of a bio-inspired hybrid multifunctional drug delivery system based on diatom microalgae. The microalgae’s surface was chemically functionalized with hybrid vitamin B_12_-photoactivatable molecules and the materials further loaded with highly active rhenium(I) tricarbonyl anticancer complexes. The constructs showed enhanced adherence to colorectal cancer (CRC) cells and slow release of the chemotherapeutic drugs. The overall toxicity of the hybrid multifunctional drug delivery system was further enhanced by photoactivation of the microalgae surface. Depending on the construct and anticancer drug, a 2-fold increase in the cytotoxic efficacy of the drug was observed upon light irradiation. The use of this targeted drug delivery strategy, together with selective spatial–temporal light activation, may lead to lower effective concentration of anticancer drugs, thereby reducing medication doses, possible side effects and overall burden for the patient.

## 1. Introduction

Targeted drug delivery for the confined treatment of colonic diseases is becoming increasingly important to address locally life-threatening disorders like colorectal cancer (CRC). Colon-focused drug delivery (CFDD) permits direct treatment at the disorder site, lowers drug dosing and decreases the chance of systemic side-effects. Indeed, most available chemotherapeutics are poorly tolerated by patients and induce severe side effects that are unbearable in many cases. CFDD is a strategy actively pursued to address inflammatory bowel diseases [1,2,3] and most importantly CRC. Indeed, despite advances in diagnostic and therapeutic modalities, CRC is the third most commonplace cause of cancer-related deaths worldwide. Generally, CRC originates from the inner wall of the colorectal epithelium, develops as a polyp and finally spreads by invading nearby lymph nodes and other organs. As pointed out in recent reviews, issues like the path of the gastro-intestinal (GI) tract, dynamic pH changes and the inability to discern healthy tissues from cancerous ones must be overcome in order to develop effective materials for CFDD treatment [4,5,6].

Different porous materials such as zeolites, nanoparticles, dendrimers/polymers, nano-hydrogels or metal organic frameworks were shown to be prospective tools in the field of drug delivery systems (DDSs) and cancer-targeted treatments [7,8,9,10,11,12,13,14,15,16,17]. In terms of material design for colon-focused drug delivery, several approaches have been described. Salleh et al., e.g., evaluated the performance of gelatin-coated type Y zeolite for the controlled release of the anticancer drug zerumbone [18] encapsulated in its pores, showing sustained and prolonged drug release over 24 h [19]. Li and coworkers addressed CFDD via a nano-in-micro dual drug delivery platform composed of halloysite nanotubes and a pH-responsive polymer. This clay mineral was loaded with atorvastatin and celecoxib, which were released only at pH 7.4 and effectively inhibited colon cancer cell proliferation [20]. Xu et al. recently presented up-conversion nanoparticles (UCNPs) to trigger cancer immunotherapy in CRC by NIR-induced photodynamic therapy (PDT) to directly destroy tumor cells and to stimulate immune responses by triggering the maturation of dendritic cells and secretion of cytokines [21]. Others have used gold- [22] and folate-coated nanoparticles for active CFDD of nanotherapeutics [23]. Finally, different authors have used reactive oxygen species (ROS)-responsive nanoplatforms and polymers as drug delivery systems with excellent results [6].

**Figure 1:**
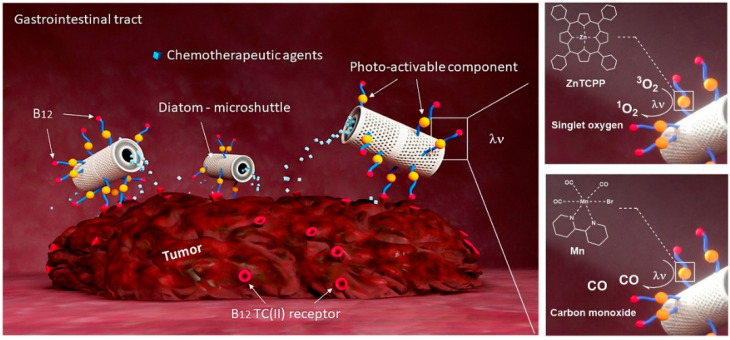
**Left**. Concept showing the bio-inspired hybrid multifunctional drug delivery system described in this study. Natural diatom microalgae (DEMs) are functionalized at their surface with photoactivatable elements (orange spheres) linked to vitamin B_12_ (red spheres) acting as the outer tumor-targeting layer. The construct can be loaded with a chemotherapeutic drug (light blue squares) to be selectively delivered to the tumor site in the intestinal tract. Once DEMs are bound to the tumor mass, the chemotherapeutic drug is slowly released. In addition to that, the micro-capsules can be photoactivated to generate a toxic CO or ^1^O_2_, sensitizing the tumor to the treatment. **Right**. Photo-induced chemical reactions at the surface of the diatom microalgae (*vide infra* for more details).

We have recently begun to develop CFDD materials based on environmentally sustainable, abundant and inexpensive diatom microalgae (DEMs) [24] with the aim of designing an innovative structure able to simultaneously target the tumor site and release loaded chemotherapeutics in its immediate proximity [25]. DEMs, as the fossil frustules of diatoms, recently gained attention for their use in drug delivery due to their biocompatibility and ability to shuttle and slowly release different drugs [26,27,28,29]. Diatoms are enclosed in a three-dimensional, highly ordered silica shell (called frustule) and represent an inexpensive and well-engineered source of microporous silica [30,31,32,33]. Our original proof of concept relied on the chemical functionalization of the diatom microalgae’s surface with vitamin B_12_, which allowed specific binding of the material to CRC cells and, therefore, discrimination between healthy and diseased tissues. The next step in our concept is illustrated in Figure 1. This bio-inspired hybrid multifunctional drug delivery system is modified by the introduction of photoactivatable units (orange spheres in Figure 1), which are envisioned to act in concert with the loaded chemotherapeutic drugs (light blue squares) in order to (a) sensitize the tumor and (b) lower the overall drug dose needed for effective treatment.

For this purpose, two complex bio-vehicles were synthetized. In both cases, the outer layer of the microalgae was still composed of cobalamin (vitamin B_12_, red spheres in Figure 1). The inner layers of the constructs were built with either a chemical photosensitizer (for photodynamic therapy, PDT) or a photo-triggered CO-releasing molecule (see right inserts in Figure 1). Photodynamic therapy is a minimally invasive treatment incorporating three different components: a light source, a chemical photosensitizer (PS) and tissue oxygen. This process aims to generate endogenously highly toxic singlet oxygen (^1^O_2_) and reactive oxygen species (ROS) upon light irradiation [34]. The produced excited-state singlet oxygen species induce cell apoptosis in tumors [35,36]. The advantages of this technique are the spatio-temporal controlled activity allowed by the photoactivation and the potential to be combined with other therapeutic treatments as chemotherapy, surgery, radiotherapy or immunotherapy [37]. As chemical photosensitizer for our hybrid multifunctional drug delivery system, we chose porphyrins, as they absorb at several wavelengths in the visible region (*Soret* band in the blue and *Q*-bands in the red) and show long-lived triplet states allowing high ^1^O_2_ quantum yields [38,39,40,41]. Furthermore, coordination of metal ions such as Zn^2+^, Ga^3+^ or Si^4+^ allows tuning ^1^O_2_ generation capacities of porphyrins, giving long excited-state lifetimes and permitting high singlet oxygen yields [42]. As a photo-triggered CO-releasing molecule, we selected a (2,2′-bipyridine)-3,4′-dicarboxylic acid manganese tricarbonyl complex. Such types of complexes (*vide infra* for detailed chemistry) are well known to decompose if photo-irradiated, liberating in the process carbon monoxide. CO, in turn, is known to exhibit antiproliferative and cytotoxic effects in cancer treatments [43,44,45,46,47]. In this contribution, we describe the preparation of these new bio-inspired hybrid multifunctional drug delivery systems. In addition to that, we detail CRC cellular interaction of the same, their drug-loading and -releasing properties, and their effects on the proliferation of CRC HCT-116 cells in the dark and when photoactivated.

## 2. Materials and Methods

### 2.1. Chemicals and Materials

Diatomaceous earth microalgae (DEMs), in the form of Celatom^®^ Fw-14, were obtained from Applied Minerals Ltd. (Burton, Staffordshire, UK). Pyrrole was purchased from Acros Organics (Hanover Township, NJ, US) and 4-carboxybenzaldehyde from Fluka Chemicals. 1-ethyl-3-(3-dimethylaminopropyl)carbodiimide (EDC), N-hydroxysuccinimide (NHS), tetra-phenylporphyrin (TPP) and 9,10-dimethylanthracene (DMA) were purchased from TCI Europe (Zwijndrecht, Belgium). N,N-dimethylformamide (DMF), Dimethyl sulfoxide (DMSO), Sodium dithionite, Triethylamine (TEA), Vitamin B_12_ (cyanocobalamin), 1,1′-Carbonyl-di-(1,2,4-triazole) (CDT), 4,7,10-trioxa-1,13-tridecanediamine (PEG linker) and zinc acetate dihydrate were delivered by Sigma-Aldrich (Buchs, Switzerland). (3-aminopropyl)triethoxysilane (APTES), H_2_SO_4_ and HCl were purchased from Honey-well Research chemicals (Bucharest, Romania). H_2_O_2_ and glycerol were purchased from Reactolab SA (Servion, Switzerland). Rhenium complexes (**1** and **2**) used as drug models were synthetized as described in a previous paper [48]. Simulated gastric fluid (SGF) was prepared as described by Coughlin et al. [49].

### 2.2. Characterization Techniques

NMR analyses were performed with a Bruker Advance III (Bruker Switzerland AG, Fällenden, Switzerland) 500 MHz, or 400 MHz. The corresponding ^1^H chemical shifts are reported relative to residual solvent protons and carbons. Mass analyses were performed either using ESI–MS on a Bruker FTMS 4.7-T Apex II in positive mode or MALDI with a Bruker UltrafleXtreme MALDI-TOF (Bruker Switzerland AG, Fällenden, Switzerland). UV-Vis spectra were measured using a Jasco V730 spectrophotometer (Echallens, Switzerland). Solid-state UV-Vis spectra were measured using a Perkin Elmer UV/VIS/NIR Spectrometer Lambda 900 with a Perkin Elmer 150 mm Int. Sphere (Schwerzenbach, Switzerland). The dry powder samples (ca. 5 mg) were dispersed between two quartz slits and stacked before being analyzed. IR spectra were measured using a Perkin Elmer FTIR Frontier Serie 99155 (Schwerzenbach, Switzerland) equipped with a PIKE TECHNOLOGIES GladiATRTM(OPUS 7.5 software). Preparative HPLCs were performed with a Merck Hitachi L-7000 (Chromaster system manager), which comprises a Pump L-7100 and a UV-Detector L-7400. A column Macherey-Nagel Nucleodur C18 HTec (5 μm particle size, 110 Å pore size, 250 × 21 mm, Oensingen, Switzerland) was used. Aqueous trifluoroacetic acid 0.1% solution and pure methanol were respectively used as solvents (A) and (B). Vitamin B_12_ and TCPP derivatives were purified using the following HPLC gradients: 0–5 min (75% A), 5–35 min (75% A → 0% A), 35–45 min (100% B) or 0–5 min (50% A), 5–30 min (50% A → 0% A), 30–45 min (100% B), the flow rate set to 5 mL min^−1^ and the compounds detected at 320 nm. For the analytical HPLCs, a Macherey–Nagel Nucleodur C18 HTec (5 μm particle size, 110 Å pore size, 250 × 4.6 mm, Oensingen, Switzerland) was used. Aqueous trifluoroacetic acid 0.1% solution and pure methanol were respectively used as solvents (A) and (B). The compounds were separated using the following gradient: 0–5 min (75% A), 5–35 min (75% A → 0%A), 35–45 min (100% B) or 0–5 min (50% A), 5–30 min (50% A → 0% A), 30–45 min (100% B), the flow rate set to 0.5 mL min^−1^ and the compounds detected at 320 nm. Scanning Electron Microscopy (SEM) pictures were recorded using a Tescan Mira3 LM FE SEM (TESCAN GmbH, Dortmund, Germany). Samples were coated with 4 nm to 10 nm platinum, depending on the analysis, and recorded at 10.0 kV under high vacuum 6 × 10^−4^ Pa. Measurements of Inductively Coupled Plasma with Optical Emission Spectroscopy (ICP-OES) were performed with a Perkin Elmer Optima 7000 DV (Schwerzenbach, Switzerland). The zeta potential was measured with a DelsaMax PRO device from Beckmann Coulter (Nyon, Switzerland) using the Smoluchowski mode. Clean hydroxylated diatoms (OH-DEMs), aminated diatoms (NH_2_-DEMs), B_12_-TCPP-DEMs or B_12_-ZnTCPP-DEMs were diluted in a PBS buffer solution to 0.01 wt% and sonicated before proceeding with the measurement.

### 2.3. Density Functional Theory (DFT) Calculations

All computations were performed with the Gaussian 09 programs on the DALCO Cluster of the University of Fribourg. The procedure of Minenkov et al. [50] was used, and different combinations of functional and basis sets were tried. Geometry optimizations were performed employing a solvent continuum dielectric model for water. The hybrid-GGA (GGA = generalized gradient approximation) functional M06 and B3LYP were used in combination with the standard LanL2DZ basis sets for the optimization of B_12_-TCPP and B_12_-Mn, respectively. For the spin state of the molecules (singlet state in all cases), the default spin formalism was followed in the calculations, and default Gaussian 09 values were adopted for the numerical integration grids, self-consistent-field (SCF) and geometry optimization convergence criteria. Geometries were optimized without symmetry restrictions. The nature of the stationary points was checked by computing vibrational frequencies in order to verify true minima. No negative frequencies were observed for the reported values. The calculated molecular geometries were visualized using GaussView.

### 2.4. Syntheses

#### 2.4.1. 5,10,15,20-Tetrakis (4-Carboxyphenyl) Porphyrin (TCPP) 

TCPP was synthetized via the monopyrrole tetramerization method originally described by Adler et al. and slightly modified [51,52]. First, 1.5 g of 4-carboxybenzaldehyde (10 mmol) was added to 50 mL acetic acid, stirred and heated to 80 °C for better dissolution of the aldehyde. Afterward, distilled pyrrole (0.7 mL, 10 mmol) was added, and the reaction mixture was brought to reflux and stirred for 2 h. After the reaction mixture was allowed to cool to room temperature, the reaction flask was placed in the fridge to induce precipitation of the porphyrin. Vacuum filtration of the reaction mixture, washing the residue with DCM (5 times 50 mL) and recrystallization permitted the isolation of a dark purple solid (1.1 g, 1.4 mmol, 55% yield), which was dried under vacuum in the oven. ^1^H NMR (400 MHz, DMSO-d6) δ(ppm) = −2.93 (s, 2 H), 8.32–8.36 (d, 8 H), 8.37–8.40 (d, 8 H), 8.85 (s, 8 H) (ESI Figure S1), which was consistent with previous studies [53]; HR-ESI-MS (ESI+) (*m*/*z*): [M + H]^+^ = 791.0, calculated for C_48_H_30_N_4_O_8_ = 790.21; UV-Vis (in MeOH, λmax, nm): 415, 512, 546, 588.5, 645.

#### 2.4.2. Vitamin B_12_ Derivative B_12_-1 

Cyanocobalamin was modified by pegylation on the 5′-hydroxylic function of the ribose moiety by slight modification of published procedures [54,55]. Briefly, 100 mg of reduced B_12_ (0.0739 mmol) was mixed with 75 mg of CDT in 3 mL of DMSO and stirred overnight. The product was then precipitated in ethyl acetate (150 mL) and centrifuged for 10 min at 6000 rpm in order to recover a red precipitate. The dried precipitate was resolubilized in 1 mL anhydrous DMF, and 100 µL of 4,7,10-trioxa-1,13-tridecanediamine in 1 mL anhydrous DMF was added and stirred for 24 h. The mixture was precipitated in 3:1 ether:ethyl acetate (150 mL) and centrifuged for 10 min at 6000 rpm before being purified by preparative HPLC. The fraction corresponding to the pegylated B_12_ (B_12_-1) was evaporated under vacuum and further lyophilisated to recover a pure dried red powder.

#### 2.4.3. Vitamin B_12_ Derivative B_12_-TCPP

In the dark and under a nitrogen atmosphere, to a solution of TCPP (25.5 mg, 3.23 × 10^−5^ mol) in 3 mL anhydrous DMF, EDC hydrochloride (12.3 mg, 6.45 × 10^−5^ mol) and NHS (7.4 mg, 6.45 × 10^−5^ mol) were added. After stirring for 1 h at room temperature, B_12_-1 (34.4 mg, 2.15 × 10^−5^ mol) in 1 mL anhydrous DMF and 20 µL TEA were added and the reaction mixture was stirred for 3 days in the dark under argon atmosphere. Afterward, the product was precipitated in 3:1 ether:ethyl acetate (150 mL) and centrifuged for 10 min at 6000 rpm before being purified by preparative HPLC (7.7 mg, 3.23 × 10^−6^ mol, 15% yield). ^1^H NMR (500 MHz, methanol-d4) δ(ppm) = 0.42 (s, 3 H), 1.05 (s, 3 H), 1.19 (d, J = 6.41 Hz, 4 H), 1.26–1.37 (m, 15 H), 1.80 (s, 3 H), 2.22 (d, J = 9.31 Hz, 6 H), 2.33 (d, J = 7.78 Hz, 2 H), 2.41–2.57 (m, 17 H), 2.79 (m, 1 H), 3.02 (s, 1 H), 3.14 (m, 1 H), 3.56–3.62 (m, 6 H), 3.64–3.76 (m, 14 H), 4.06–4.17 (m, 4 H), 4.32 (m, 1 H), 4.47 (d, J = 9.46 Hz, 1 H), 4.59 (d, J = 11.60 Hz, 1 H), 5.91 (s, 1 H), 6.18 (d, J = 2.75 Hz, 1 H), 6.52 (s, 1 H), 7.08 (s, 1 H), 7.19 (s, 1 H), 8.26–8.29 (m, 2 H), 8.33 (d, J = 6.56 Hz, 8 H), 8.47 (d, J = 8.24 Hz, 6 H) 8.85 (br. s., 8 H) (ESI Figure S2); MALDI-TOF: *m*/*z* = 2371.35, calculated for C_122_H_138_CoN_20_O_25_P^−^ = 2372.92; UV-Vis (in MeOH, λmax, nm): 360, 415, 512, 546, 588.5, 645.

#### 2.4.4. B_12_-ZnTCPP 

To a solution of B_12_-TCPP (3 mg, 1.26 × 10^−6^ mol) in 3 mL MeOH, 0.5 mg Zn(OAc)_2_∙2H_2_O were added, and the reaction mixture was stirred under reflux for 1 h. Afterward, the reaction mixture was allowed to cool to room temperature. Vacuum filtration and washing the residue several times with water allowed removing excess zinc and permitted the isolation of a brown-red solid that was dried under vacuum in the oven. UV-Vis (in MeOH, λmax, nm): 360, 423.5, 517, 560, 601.

#### 2.4.5. TCPP Derivative TCPP-1

To a solution of TCPP (30 mg, 3.8 × 10^−5^ mol) in 5 mL anhydrous DMF, EDC hydrochloride (9.1 mg, 4.75 × 10^−5^ mol) and NHS (5.5 mg, 4.75 × 10^−5^ mol) were added. After stirring for 2 h at room temperature, 10 µL of 4,7,10-trioxa-1,13-tridecanediamine and TEA(20 µL) were added and the reaction mixture was stirred for 3 days in the dark under argon atmosphere. The product was precipitated in 3:1 ether:ethyl acetate (160 mL) and centrifuged for 10 min at 6000 rpm before being purified by preparative HPLC (8 mg, 8.06–6 mol, 21% yield). ^1^H NMR (400 MHz, DMSO-d6) δ(ppm) = −2.88 (br. s., 2 H), 1.76–1.85 (m, 2 H), 1.92 (quin, J = 6.63 Hz, 2 H), 2.84–2.93 (m, 2 H), 3.52 (t, J = 6.05 Hz, 4 H), 3.54–3.57 (m, 2 H), 3.58–3.62 (m, 8 H), 7.62 (br. s., 2 H), 8.24–8.47 (m, 16 H), 8.77 (t, J = 5.56 Hz, 1 H), 8.86 (s, 8 H) (ESI Figure S3); HR-ESI-MS (ESI+) (*m*/*z*): [M + H]^+^ = 993.3, [M + 2H]^2+^/2 = 497, calculated for C_58_H_52_N_6_O_10_ = 993.09; UV-Vis (in MeOH, λmax, nm): 415, 512, 546, 588.5, 645.

#### 2.4.6. ZnTCPP-1

To a solution of 10 mg TCPP (1.0 × 10^−5^ mol) in 5 mL DMF, 2.65 mg Zn(OAc)_2_∙2H_2_O (1.2 × 10^−5^ mol) were added, and the reaction mixture was stirred under reflux for 2 h. Afterward, the reaction mixture was allowed to cool to room temperature. Vacuum filtration and washing the residue several times with water allowed removing excess zinc and permitted the isolation of a dark green solid, which was dried under vacuum in the oven (10 mg, 9.47 × 10^−6^ mol, 94.7% yield). UV-Vis (in MeOH, λmax, nm): 423.5, 560, 601.

#### 2.4.7. fac-[Mn(I)Br(CO)_3_(4,4-carboxyl-2,2-bipyridin)] (Mn)

The manganese(I) photoCORM complex was synthetized by slight modification of a published procedure [56]. Bromopentacarbonylmanganese(I) (150 mg, 0.55 mmol, 1 equivalent with 1.1 equivalent of the 2,2′-Bipyridine-4,4′-dicarboxylic acid (148 mg, 0.61 mmol) was stirred in THF (20 mL) under dark conditions overnight. Afterward, the mixture was filtered and the supernatant containing the complex (Mn) was dried under vacuum. Mn was then purified via HPLC before being evaporated to dryness in a lyophilizer. An orange-yellow solid was recovered. Yield 67.9 mg (26%). UV/Vis spectrum in methanol solution: λmax = 410 (ESI Figure S12); FTIR (ATR, cm^−1^): ν_C≡O_ = 2026, 1943, 1901, 1733 (ESI Figure S9). ESI-MS (pos^+^): [M-Br]^+^ = 383.01, calculated for C_15_H_8_MnN_2_O_7_ = 382.97. All other analytical data are in agreement with what was reported previously [56].

#### 2.4.8. Vitamin B_12_ Derivative B_12_-Mn 

Cyanocobalamin was modified by pegylation on the hydroxylic function of the ribose part (5′-OH) of the molecule as previously described [25,57]. The pegylated B_12_ was then reacted with the manganese complex Mn to give the B_12_-Mn. For this purpose, 1.6 mg (0.0035 mmol, 1.5 equivalent) of Mn were activated in MES buffer pH 5.5 with 46 µL EDC during 30 min in the dark before 17 mg of NHS were added for another 30 min and finally 3.7 mg (0.0023 mmol, 1.0 equivalent) of pegylated B_12_ was poured dropwise in 1 mL MES buffer pH 5.5. For the next 5 h, 15 mg of EDC were added each hour. Two hours after the last EDC addition, 50 µL of TEAwas added to the mixture that was allowed to react overnight in the dark. The crude was finally purified by preparative HPLC. Each manipulation was done in diminished light due to the photo-sensitivity of Mn. Yield 3.7 mg (79%). ^1^H NMR (400 MHz, MeOD-[d4]): δ = 9.11 (t, J = 5.5 Hz, 2 H), 8.88 (d, J = 7.4 Hz, 2 H), 8.16 (d, J = 4.3 Hz, 2 H), 7.79 (t, J = 5.64 Hz, 1 H) *, 7.23 (s, 1 H), 6.98 (s, 1 H), 6.42 (s, 1 H), 6.19 (d, J = 2.5 Hz, 1 H), 6.02 (s, 1 H), 4.77–4.72 (m, 2 H), 4.58 (d, J = 10.0 Hz, 1 H), 4.22–4.16 (m, 3 H), 3.87 (q, J = 5.33 Hz, 1 H), 3.63–3.60 (m, 10 H), 3.55 (t, J = 5.47 Hz, 2 H), 3.51 (q, J = 1.65 Hz, 1 H), 3.49 (s, 1 H), 3.46 (s, 1 H), 3.38 (s, 1 H), 3.30–3.12 (m, 8 H), 2.93–2.86 (m, 1 H), 2.80–2.73 (m, 2 H), 2.64–2.54 (m, 4 H), 2.53–2.39 (m, 14 H), 2.28 (s, 6 H), 2.08–1.93 (m, 8 H), 1.91–1.74 (m, 12 H), 1.74–1.65 (m, 2 H), 1.55 (s, 1 H), 1.52 (s, 1 H), 1.46 (s, 3 H), 1.53 (d, 1 H), 1.34 (s, 3 H), 1.26 (d, J = 6.3 Hz, 3 H), 1.22 (s, 3 H), 1.05 (s, 3 H), 1.01–0.88 (m, 2 H), 0.40 (s, 3H) ppm. * amide, disappear with proton exchange (ESI Figure S4); UV/Vis spectrum in methanol solution: λmax = 361, 415, 519, 551 (ESI Figure S13); FTIR (ATR, cm^−1^): ν_C≡O_ = 2043, 1961, 1944 (ESI Figure S10).

### 2.5. DEMs Functionalizations

The isolation, purification and surface activation of DEMs were done as previously described by Delasoie et al. [25]. The aminated DEMs surface was generated by APTES condensation.

#### 2.5.1. B_12_-TCPP-DEMs

**Figure 2:**
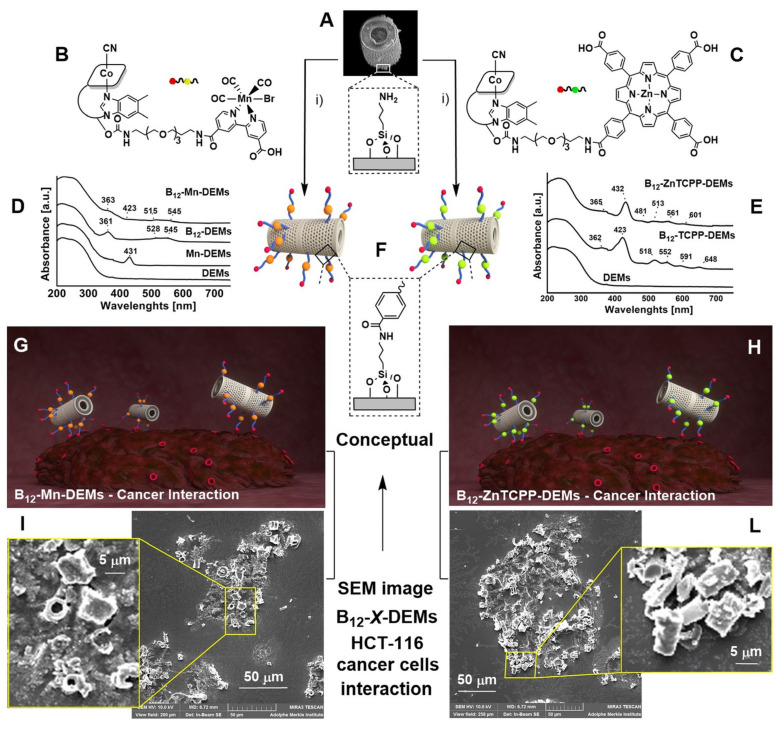
(**A**) SEM image of one raw diatom piece. (**B**,**C**) Structures of B_12_-Mn and B_12_-ZnTCPP, respectively. (**D**,**E**) Solid-state UV-Vis spectra of different functionalized DEMs. (**F**) Conceptual drawing of functionalized B_12_-*X*-DEMs (*X* = Mn or ZnTCPP) and detail of molecular bonding at the surface of microalgae. (**G**,**H**) Conceptual images of B_12_-Mn-DEMs and B_12_-ZnTCPP-DEMs interacting with a tumor mass. (**I**,**L**) SEM images of B_12_ derivatized DEMs attached to HCT-116 colorectal cancer cells and representative magnification.

To a solution of B_12_-TCPP (2.5 mg, 1.05 × 10^−6^ mol) in 1 mL anhydrous DMF, EDC hydrochloride (0.4 mg, 2.1 × 10^−6^ mol) and NHS (0.26 mg, 2.1 × 10^−6^ mol) were added. After stirring for 1 h in the dark at room temperature, ≈10 mg of aminated DEMs in 0.2 mL anhydrous DMF and TEA (20 µL) were added and the reaction mixture was stirred for 3 days in the dark at room temperature. Afterward, the functionalized DEMs were separated by centrifugation and washed several times with MeOH and water, yielding a slightly reddish powder. The material was characterized by UV solid-state (Figure 2) and zeta potential. 

#### 2.5.2. B_12_-ZnTCPP-DEMs

B_12_-TCPP-DEMs in MeOH were stirred under reflux in the presence of Zn(OAc)_2_∙2H_2_O (1 mg) for 1 h. Afterward, the functionalized DEMs were separated by centrifugation and washed several times with MeOH and water to yield a slightly brownish powder. The material was characterized by UV solid-state (Figure 2) and zeta potential.

#### 2.5.3. Mn-DEMs

In order to functionalize the aminated DEMs with Mn, 0.5 mg of Mn were dissolved in 1 mL DMF with 1 mg of EDC under diminished light and stirred for 30 min. Then, 1 mg of NHS was added and let react for the next 30 min. After direct addition of ca. 10 mg of aminated DEMs and 20 µL TEA, the mixture was reacted overnight at RT in the dark. The mixture was then centrifuged and the supernatant discarded, and this was repeated with 10 mL DMF and 10 mL methanol thrice. The sample was finally dried under vacuum. The recovered powder was denoted as Mn-DEMs. 

#### 2.5.4. B_12_-Mn-DEMs 

To achieve the functionalization of aminated DEMs with B_12_-Mn, typically 0.8 mg of B_12_-Mn was added to 0.15 mg of EDC chloride and 0.10 mg of NHS in 1 mL DMF for 1 h diminished light. Then, 2 mg of DEMs in 0.5 mL DMF was added to the mixture. Twenty microliters of TEA were added directly after. The mixture was reacted at RT in the dark overnight. The mixture was then centrifuged and the supernatant discarded. The recovered powder was washed with 20 mL DMF and with 20 mL methanol thrice and finally dried under vacuum overnight. The recovered powder was denoted as B_12_-Mn-DEMs.

### 2.6. Photodynamic Measurements

For fluorescence quantum yield and lifetime measurements, diluted solutions of ZnTCPP, ZnTCPP-1 and B_12_-ZnTCPP in DMSO were prepared in 1 cm quartz cuvettes. Fluorescence spectra were recorded with an FS5 Spectrofluorometer from Edinburgh Instruments (General Microtechnology & Photonics, Renens, Switzerland). Fluorescence quantum yields were measured with the same instrument equipped with an integrating sphere. The fluorescence lifetime of the samples was determined with a time-correlated single-photon-counting (TCSPC) LifeSpec II instrument from Edinburgh Instruments General Microtechnology & Photonics, Renens, Switzerland) equipped with an EPL-405 picosecond pulsed diode laser from Edinburgh Photonics (General Microtechnology & Photonics, Renens, Switzerland). The detection was set to emitted photons at a wavelength of 660 nm. Both quantum yields and lifetime data were elaborated with Fluoracle® software (2015) provided by Edinburgh Instruments.

Singlet oxygen quantum yield. ($\Phi_{\Delta }$) were determined via assessment of light absorption decrease based on the oxidation of 9,10-dimethylanthracene (DMA) generating an endoperoxide in a [4 + 2]-reaction. Tetraphenylporphyrin (TPP) in DMSO ($\Phi_{\Delta }^{R}$= 0.52) was used as reference to calculate $\Phi_{\Delta }$ for the PS [58]. A DMA solution (1.45 × 10^−4^ M in DMSO) was mixed with the PS in a 1 cm quartz cuvette and bubbled with oxygen for 5 min. The absorbance of the reaction mixture was taken at 401 nm. The cuvette was irradiated by a 100 W LED-light at 420 nm (LUMOS 43 from Atlas Photonics), and the decrease in the absorbance of DMA at 401 nm was followed. The measurements were done in triplicate. The kinetics of DMA photo-oxidation permits one to calculate the singlet oxygen generation of the PS. The following equation is used:

(1) $$\Phi_{\Delta }^{S}=\Phi_{\Delta }^{R}\frac{K^{S}I^{R}}{K^{R}I^{S}}$$

where $K^{R}$ and $K^{S}$ are the slopes of the kinetic plot of the difference in absorbance vs. irradiation time of DMA photo-oxidized by the reference and sample, respectively; $I^{R}$ and $I^{S}$ the total light intensities absorbed by the reference and sample and $\Phi_{\Delta }^{S}$ the singlet oxygen quantum yield of the sample. Light intensities were calculated according to the following equation:

(2) $$I=I_{0}\;\left(1-10^{-A\left(\lambda =420\;\mathrm{nm}\right)}\right)$$

where I is the absorbed light intensity from the sample, $I_{0}$ the light intensity from the light source and A (λ=420 nm) the absorbance of the photosensitizer at the excitation wavelength.

### 2.7. Kinetic and Equivalent CO Released by Photoirradiation

For both Mn and B_12_-Mn complexes, the kinetic of CO release was evaluated by monitoring the spectral changes at a specific wavelength (410 and 365 nm respectively) by UV/Vis spectroscopy. For this purpose, the complexes were firstly dissolved in DMSO (1% *v*/*v*, final concentration) before being diluted in 0.1 M PBS. Samples were irradiated with a 420 nm light. Then, the equivalent of CO release was evaluated under the conditions of myoglobin assay for all complexes. The solutions Mn (15 µM) and B_12_-Mn (10 µM), measured in 0.1 M PBS buffer at pH 7.4, 10 mM dithionite, with 3 equivalent of myoglobin (respectively 45 and 30 µM), under argon, were irradiated with a 420 nm light. The stability of each compound in the presence of dithionite was evaluated prior to each experiment, and none of them showed spontaneous CO release. The concentration of Myoglobin-CO complex c(Mb-CO) formed through time due to CO release from the photoCORMs upon irradiation at 420 nm was monitored, and the CO release equivalent was calculated as described by Atkin et al. [59]. Due to its poor solubility, Mn was previously dissolved in DMSO (1% *v*/*v*, final concentration).

### 2.8. Resistance of B_12_-X-DEMs in Simulated Digestive Fluids

The test was performed in simulated gastric fluid (SGF). For this purpose, ca. 4 mg of each B_12_-*X*-DEMs sample was digested in SGF (6 mL) for 2 h before the solid residue and the supernatant were recovered. The supernatant was diluted to 10 mL with HNO_3_ 2% (*v*/*v*). The solid fraction was digested for a second time in 2 mL piranha solution (H_2_SO_4_:H_2_O_2_ 7:1) overnight before being diluted to 10 mL with deionized water. All the samples were analyzed by ICP–OES (Co monitoring). The result was calculated as a mass ratio of the B_12_ equivalent compared to the total amount of sample.

### 2.9. Drug Loading and Release from DEMs

Around 2 mg of the rhenium complexes used as model drugs (**1** or **2**, *vide infra*) was weighed and dissolved in 20 μL of DMSO, and then 10 μL of this solution of 100 mg/mL was diluted (100 fold) to 1000 μL with aqueous buffer PBS pH 7.4 (1% *v*/*v*, DMSO), giving the first standard solution (S1, 1.0 mg mL^−1^). Dilutions of S1 in PBS pH 7.4 (1% *v*/*v*, DMSO) gave the standards used for the calibration curves (5 standards, R^2^ > 0.9). The standards and samples were analyzed by UV-Vis spectroscopy. For each measurement, the sample was quickly vortexed before being transferred for UV-Vis measurement. The drug loadings (DL) were performed by weighing around 10 mg of the drug and the same amount of DEMs in an Eppendorf. Afterward, 1 mL of acetone was added to obtain a 10 mg mL^−1^ drug solution. The samples were shaken on the plate for 48 h. The mixtures were then centrifuged and the supernatant removed. A quick wash was performed by adding 500 μL of acetone, centrifuging 5 s and removing supernatant. The samples were then evaporated under argon to dryness. Drug release experiments were performed by adding 1000 μL of PBS pH 7.4 (1% DMSO) to the drug-loaded DEMs samples. Samples of 100 µL were taken at each time point and replaced with fresh buffer. The concentrations of these samples were evaluated by UV-VIS spectroscopy by monitoring the absorption of the complexes at specific wavelengths. The values for the cumulative drug release in percentage were calculated from the linear regressions previously established. The graphs were plotted using Origin 7.5. The DL were calculated as the mass percentage (wt%) of drug over the sum of drug and DEMs.

### 2.10. Cytotoxicity Assessment

Rhenium anticancer complexes **1** and **2** were freshly dissolved first in DMSO and then in RPMI 1640 medium (Merck, Munich, Germany), while the other analyzed compounds were immediately dissolved in RPMI 1640 medium and used for bioactivity assessments. In vitro cytotoxicity in terms of antiproliferative effects was determined by (3-(4,5-dimethylthiazol-2-yl)-2,5-diphenyltetrazolium bromide (MTT) assay [60] on colorectal cancer HCT116 cell line, obtained from American Type Culture Collection (ATCC). The cells, cultured in the complete RPMI 1640 medium as a monolayer (1 × 10^4^ cells per well), were incubated with test compounds for 48 h in humidified atmosphere of 95% air and 5% CO_2_ at 37 °C, and the MTT assay was carried out two times in four replicates. The extent of MTT reduction was measured spectrometrically at 540.0 nm using *Tecan Infinite 200 Pro* multiplate reader (Tecan Group Ltd., Männedorf, Switzerland), and the cell survival was expressed as a percentage of the control arbitrarily set to 100%. Cytotoxicity was expressed as the concentration of the compound inhibiting cell growth by 50% (IC_50_) in comparison to untreated control. For the photoactivation experiment, Osram L30W/840 cool white light Lumilux (Munich, Germany) has been used two times for 10 min, immediately upon the addition of compounds to the cells and 1 h after.

## 3. Results and Discussion

### 3.1. Synthesis and Characterization of Surface Coating Molecules

**Scheme 1:**
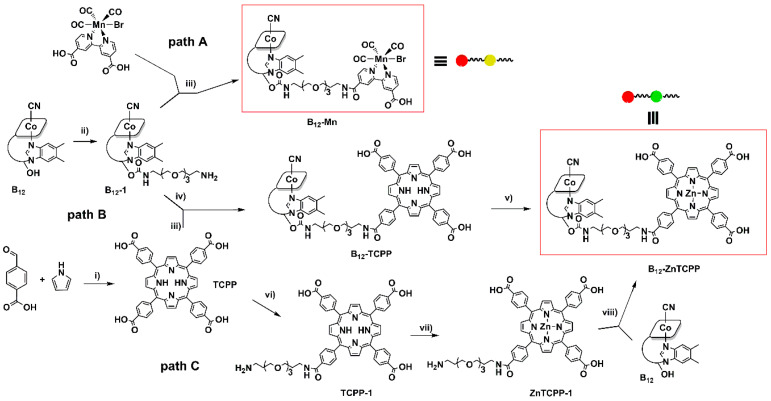
Reaction steps for the synthesis of B_12_-Mn and B_12_-ZnTCPP. Reagents and conditions: (i) 2 h, acetic acid under reflux; (ii) CDT, 24 h, DMSO, then PEG, 24 h, anhydrous DMF; (iii) EDC/NHS, 1 h, anhydrous DMF, diminished light, RT; (iv) 72 h, anhydrous DMF, RT; (v) Zn(OAc)_2_∙2·H_2_O, 1 h, MeOH under reflux; (vi) EDC/NHS, 2 h, anhydrous DMF, RT; (vii) Zn(OAc)_2_∙2·H_2_O, 1 h, MeOH under reflux; (viii) CDT, DMSO, 12 h/anhydrous DMF, 24 h.

**Figure 3:**
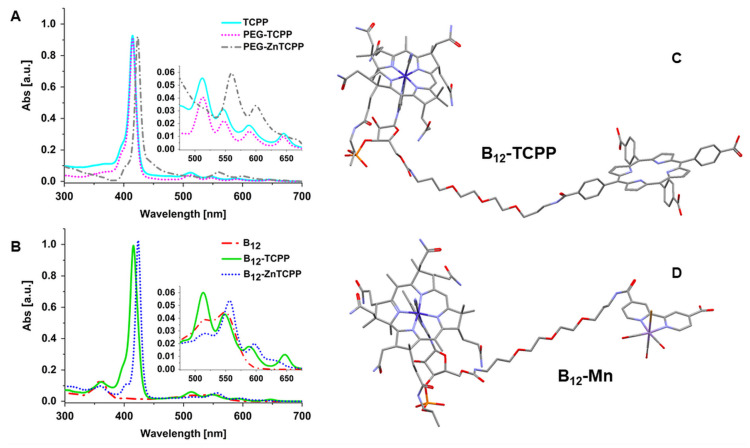
(**A**,**B**) represent the UV-Vis absorption spectra of TCPP, PEG-TCPP, PEG-ZnTCPP and B_12_, B_12_-TCPP, B_12_-ZnTCPP, respectively; (**C**,**D**) show optimized structures of B_12_-TCPP and B_12_-Mn, respectively, calculated in silico by Density functional theory (DFT) (polarizable continuum model with water as solvent). C-atoms are grey, N-atoms blue, O-atoms red, P is orange and Co dark blue. H-atoms are omitted for clarity.

Vitamin B_12_ (tumor-targeting agent) surface-coated molecules bearing either a (2,2′-bipyridine)-3,4′-dicarboxylic acid manganese tricarbonyl complex (photoCORM, B_12_-Mn) or a zinc of 5, 10, 15, 20-tetrakis(4-carboxyphenyl)porphyrin molecule (PDT agent, B_12_-ZnTCPP) were prepared according to the synthetic procedure illustrated in Scheme 1. Vitamin B_12_ (represented by a simplified structure) was first modified with an aminated PEG-chain on the 5′-OH ribose moiety and then reacted directly with the manganese complex (Mn, giving B_12_-Mn, path A in Scheme 1) or with 5,10,15,20-tetrakis(4-carboxyphenyl)porphyrin (TCPP) respectively (path B in Scheme 1). The latter was finally metalated with zinc(II) acetate, giving B_12_-ZnTCPP. Alternatively, B_12_-ZnTCPP could be prepared by PEGylation of TCPP followed by metalation and coupling to vitamin B_12_ (path C in Scheme 1). We noted that the length of the selected PEG-chain allows vitamin B_12_ derivatives to be still recognized by the transcobalamin receptors (TC(II) and TC(II)-R), leading to increased adherence towards cancer cells [25,61,62]. Density functional theory (DFT) calculations of B_12_-Mn and B_12_-TCPP performed in water (polarizable continuum model, *vide infra*, Figure 3) indicate a distance of ca. 18 Å between the molecular units. Thus, the PEG chain does not only act as a linker but also provides a certain degree of freedom to the system. Indeed, we expected this distance to minimize steric hindrance in the final composite materials, allowing the different components to play their role with minimal interferences [63]. Once grafted onto the surface of diatom microalgae, this arrangement facilitates the role of B_12_ as the tumor-targeting agent [25]. At the same time, photoactivation of the surface allows for CO release from B_12_-Mn or ^1^O_2_ generation from B_12_-ZnTCPP respectively. Both surface-coating derivatives were characterized by standard techniques. ^1^H-NMR, visible spectroscopy and MS data are fully consistent with the proposed molecular structures of B_12_-Mn and B_12_-ZnTCPP. Particularly revealing were the ^1^H-NMR spectra of the molecules (ESI Figures S1–S4). As expected, the homotopicity of the aromatic protons of the (2,2′-bipyridine)-3,4′-dicarboxylic acid and TCPP of B_12_-Mn and B_12_-ZnTCPP, respectively, was lost upon coupling to B_12_-1 (i.e., PEGylated vitamin B_12_, see Scheme 1). In the case of B_12_-ZnTCPP, e.g., the symmetric 1:1 doublets of TCPP split into three doubles of relative intensity of 4:3:1; the latter being assigned to the 4-carboxyphenyl protons in ortho-position to the newly formed amide bond (ESI Figure S1). Metalation of B_12_-TCPP was confirmed by UV-Vis spectroscopy. Indeed, the *Soret* band of the porphyrin undergoes a bathochromic shift from 415 to 423 nm upon reaction with Zn(OAc)_2_∙2·H_2_O (Figure 3). Moreover, upon metalation, the degeneracy in the porphyrin orbital causes the coalescence of the four TCPP *Q*-bands (512, 546, 588 and 645 nm) into two *Q*-bands in B_12_-ZnTCPP (560 and 601 nm), as previously described [64,65]. The peaks at 360 nm and 517 nm were respectively attributed to the α-band and β-band of vitamin B_12_ (Figure 3).

### 3.2. ^1^O_2_ Generation and CO Release of Surface Coating Molecules

Fluorescence quantum yield ($\Phi_{fl}$), lifetime (τ_f_) and singlet oxygen (^1^O_2_) quantum yield ($\Phi_{\Delta }$) were assessed for PEG-ZnTCPP and B_12_-ZnTCPP in DMSO (Table 1). Fluorescence spectra of PEG-ZnTCPP and B_12_-ZnTCPP in DMSO show emission bands around 612 and 666 nm (ESI Figure S7), and the fluorescence quantum yields and lifetimes are similar for the two species. The results are consistent with values reported in the literature for comparable porphyrin analogs and support the hypothesis that the structural change at the periphery of the porphyrin does not drastically affect $\Phi_{fl}$ and τ_f_ since the fluorescence phenomenon arises from the inner π–electron system on the macrocycle [65,66,67]. Nevertheless, it is known that introducing electron-donating or -withdrawing groups onto porphyrin center will slightly influence the fluorescence properties [68]. Longer fluorescence lifetime and improved inter-system crossing positively influence the singlet oxygen quantum yield, but the same is affected by many other parameters such as solubility or the tendency of the fluorophore to form aggregates. However, $\Phi_{\Delta }$ does not correlate to the fluorescence lifetime and quantum yield improvement. Porphyrins can easily aggregate in solution, thereby lowering their efficiency in energy transfer, with ^3^O_2_ resulting in lower ^1^O_2_ $\Phi_{\Delta }$ [69,70]. The solubility and aggregation propensity of the zinc(II) porphyrin are substantially modified by introducing a hydrophilic PEG chain or a large and highly water-soluble structure like vitamin B_12_. The coupling of vitamin B_12_ to ZnTCPP slightly increases the singlet oxygen quantum yield by 15%. Indeed, vitamin B_12_ covalently linked to the porphyrin enhances the solubility of the structure and lowers its tendency to aggregate, thereby leading to an efficient generation of ^1^O_2_ singlet oxygen. B_12_-ZnTCPP is stable to light. We observed no decomposition of the molecule under irradiation, suggesting the great potential of B_12_-ZnTCPP in PDT. Pseudo-half-life and equivalents of CO released were measured for B_12_-Mn and its constituent photoCORM complex (Mn) [71] according to the method described by Atkin et al. (Table 1) [59]. To this end, changes in the UV-Vis spectrum at 410 and 365 nm were monitored as a function of irradiation time for Mn and B_12_-Mn, respectively (ESI Figures S18–S21). The stabilities of both complexes were demonstrated by the absence of spontaneous CO releases in the dark in the presence of sodium dithionite. Both molecules, upon light irradiation, released ca. one equivalent of CO (ESI Figures S16 and S17). 

### 3.3. Surface Functionalization, Characterization and DEMs Cellular Interaction

Surface functionalization of DEMs with B_12_-ZnTCPP and B_12_-Mn was achieved via amide-bond formation with one of the free carboxylic functions available on the molecules and the previously aminated surface of the DEMs (Figure 2). In the case of B_12_-TCPP and B_12_-ZnTCPP, the presence of free carboxylic functions on the porphyrin macrocycle in both *cis*- and *trans*-positions to the PEG arm offers the possibility of several binding modes to DEMs. Given the fact that B_12_-ZnTCPP-DEMs interact strongly with cancer cells (Figure 2) we posit either that the *trans*-4-carboxyphenyl is preferentially favored or that the system is flexible enough so that steric hindrance (i.e., in the case of *cis*-4-carboxyphenyl binding) does not play a significant role in B_12_-ZnTCPP-DEMs cell interaction.

Successful surface functionalization of diatoms microalgae was assessed via solid-state UV-Vis spectroscopy (Figure 2D,E). For B_12_-TCPP-DEMs, comparison of the spectra of the new material to the non-functionalized DEMs, shows new absorbance peaks at 362 nm (*Soret* band of vitamin B_12_), 423 nm (*Soret* band TCPP), 518, 552, 591, and 648 nm (*Q*-bands of TCPP). By further reacting B_12_-TCPP-DEMs with zinc acetate in methanol under reflux, metalation of the porphyrin centers occurred, leading to B_12_-ZnTCPP-DEMs. The solid-state UV-Vis spectrum of B_12_-ZnTCPP-DEMs compared to the non-metaled equivalent shows a bathochromic shift of the TCPP *Soret* band to 432 nm and coalescence of the *Q*-bands to two peaks at 561 and 601 nm (Figure 2E). This evidence confirmed the successful metalation of the material. Similarly, solid-state UV-Vis spectroscopy was performed to evaluate the newly synthetized material B_12_-Mn-DEMs. When comparing the DEMs spectra before and after surface functionalization with pure Mn, B_12_ and B_12_-Mn, the appearance of new peaks corresponding to each compound could be observed (Figure 2D). For the Mn-DEMs (synthetized to assess the feasibility of the surface coating), a peak at 431 corresponding to the MLCT absorbance spectrum of the Mn complex is detected. In the case of B_12_-DEMs [25], peaks at 361 nm, 521 nm and 545 nm are consistent with absorbance of pure cyanocobalamin [72]. Finally, the spectrum of B_12_-Mn-DEMs shows peaks corresponding to cobalamin at 363 nm, 515 nm and 545 nm as well as a very small peak at 423 nm attributed to the presence of Mn. The solid-state UV-Vis spectrum of B_12_-Mn-DEMs is consistent with the spectrum of B_12_-Mn in solution (Figure S13), which shows a signal at 420 nm, very low in intensity when compared to the peaks attributed to the vitamin B_12_ at 361, 521 and 545 nm.

Further direct characterization of successful DEMs surface functionalization came from zeta potential (ζ-potential) analysis of B_12_-ZnTCPP-DEMs. Zeta potential of different samples of DEMs were recorded in PBS buffer at pH 7.4. Clean hydroxylated diatoms (DEMs-OH) expressed a negative potential of −23.4 ± 0.5 mV, which shifted to positive +9.5 ± 0.4 mV upon surface amination with APTES. This positive shift is indicative of amine functions on DEMs, which can be easily protonated in the buffer [25,73,74,75]. Further functionalization with TCPP moves the ζ-potential back towards the negative value of −53.0 ± 0.4 mV, logically deriving from the presence of carboxylic acid groups. The same potential slightly increases to −48.8 ± 0.3 mV for B_12_-TCPP-DEMs and remained virtually the same following Zn metalation for B_12_-ZnTCPP-DEMs (−47.1 ± 0.2 mV).

The surface functionalization of B_12_-Mn-DEMs and B_12_-TCPP-DEMs, as well as the resistance of the surface coating in simulated gastric fluid, were assessed by inductively coupled plasma atomic emission spectroscopy (ICP-OES). Naturally occurring diatoms microalgae may contain small traces of cobalt in their frustules composition but considerable traces of manganese [76]. For example, in *Cyclotella meneghiniana* and *Stephanodiscus hantzschii*, common freshwater diatom species, zinc and manganese compete for cell wall incorporation with Mn accumulating preferentially at the girdle band of frustules [77,78]. In contrast, cobalt can only replace zinc when this ion is totally absent from the growth medium [79]. ICP-OES measurements of the cobalt content in clean DEMs indicated values below the limit of quantification (4 ppm). The difficulty of generating Co ions from cobalamin in the plasma has been reported before [80]; nevertheless, the method we used in the present study was already proven efficient to determine about 95% of the B_12_ present in a sample [25]. The B_12_-equivalent content was extrapolated from Co concentration of each sample after 2 h digestion in SGF. B_12_-Mn-DEMs showed concentrations of 450 ± 19 ppm and 131 ± 10 ppm of B_12_ for supernatant and solid residue, respectively. B_12_-TCPP-DEMs showed concentrations of 224 ± 28 ppm and 92 ± 21 ppm of B_12_ for supernatant and solid residue, respectively. Results mean that ca. 29% and ca. 41% of the surface coating remain intact after 2 h in the stomach for B_12_-Mn-DEMs and B_12_-TCPP-DEMs, respectively. We plan to coat with gel to increase performance.

Scanning electron microscopy was used to assess the adherence of B_12_-ZnTCPP and B_12_-Mn on colorectal cancer HCT-116 cells. In order to demonstrate cellular adherence, cells were treated with either unmodified (DEMs) and B_12_-functionalized diatoms under identical conditions (200 µg biomaterial mL^−1^, 1 h exposure). After intensive washes, cells exposed to B_12_-functionalized diatoms still presented and retained a large amount of micro-particles (Figure 2I,L), while in the sample of unmodified DEMs, almost all the micro-particles were removed via the washing steps. 

### 3.4. In vitro Drug Loading and Release from Diatom Microalgae

**Figure 4:**
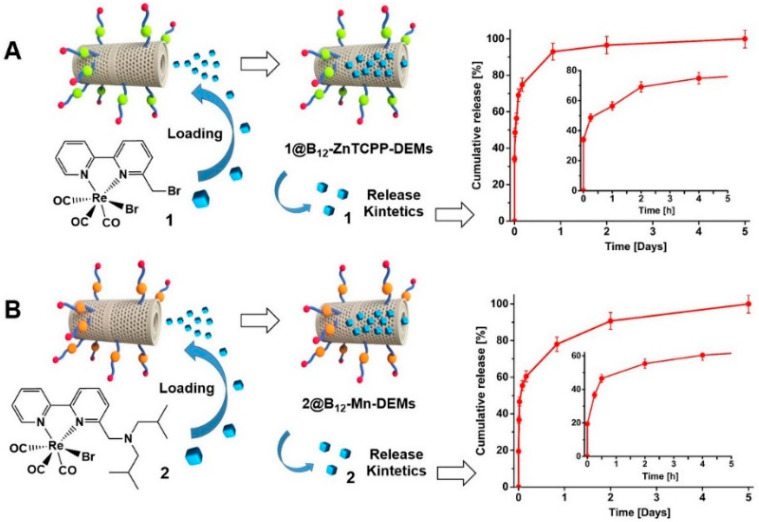
(**A**) Representative loading of B_12_-ZnTCPP-DEMs with complex **1** (giving **1**@B_12_-ZnTCPP-DEMs) and graph showing the release of **1** from B_12_-ZnTCPP-DEMs over a period of 5 days (insert shows release in the first 5 h). (**B**) Representative loading of B_12_-Mn-DEMs with complex **2** (giving **2**@B_12_-Mn-DEMs) and graph showing the release of **2** from B_12_-Mn-DEMs over a period of 5 days (insert shows release in the first 5 h).

We have recently described the anti-proliferative efficacy of a series of rhenium(I) tricarbonyl *N*-derivatized *N*-((2,2′-bipyridin)-6-ylmethyl) complexes of increasing lipophilicity against different cell lines [48]. Among the series of these compounds, the *fac*-(Re(CO)_3_Br) complexes with 6-(bromomethyl)-2,2′-bipyridine (**1** in this study, Figure 4) and *N*-((2,2′-bipyridin)-6-ylmethyl)-*N*-isobutyl-2-methylpropan-1-amine (**2**, Figure 4) showed excellent in vivo toxicity profiles and in vivo anticancer/antimetastatic efficacy and effective inhibition of angiogenesis in the zebrafish–human CRC tumor xenograft model [48]. These two complexes were selected for the present study. The molecules were successfully loaded into DEMs according to the vacuum infiltration method described by Vasani et al. [81] (giving **1**@B_12_-ZnTCPP-DEMs and **2**@B_12_-Mn-DEMs, respectively) with respective loading degrees of 3.7 (**1**) and 1.5 (**2**) wt% (Table 2). These are in the typical range of drugs loaded into DEMs biosilicas [25,26,82,83,84]. The drug–material composite selection was based on the most favorable drug-release characteristics for each combination. Following successful encapsulation, the drug release in PBS buffer pH 7.4 (1% *v*/*v*, DMSO) was initiated by the addition of solvent and monitored by UV-VIS spectroscopy over a period of 5 days in both cases. As often observed in different studies, an important initial burst release occurs during the first hours (Figure 4) [27,29]. Ca. 60% and 50% of complexes **1** and **2** were respectively released in solution after the first hour. A second more gradual and sustained release was then observed over the course of a few days. Indeed, **1** and **2** were nearly completely discharged from B_12_-ZnTCPP-DEMs and B_12_-Mn-DEMs after 2 and 5 days, respectively.

### 3.5. PDT and Cytotoxicity Assessment

**Figure 5:**
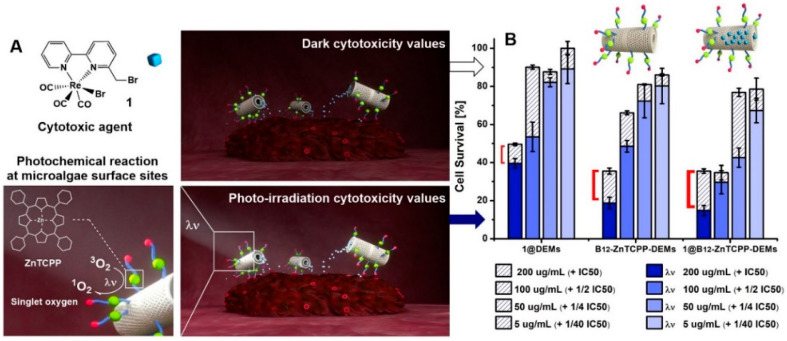
(**A**) Conceptual representation of the experiment, structure of complex **1** and detail of the photochemical reaction taking place at the microalgae surface sites (ZnTCPP-induced ^1^O_2_ generation). (**B**) Histograms representing the effects on HCT-116 colorectal cancer cells % survival by different doses of **1**@DEMs, B_12_-ZnTCPP-DEMs and **1**@B_12_-ZnTCPP-DEMs when administered in the dark (empty bars) and under the same conditions following light activation of the microalgae surface (filled colored bars). The red square brackets on the graph are meant to highlight the enhanced cytotoxicity of the material upon photo-irradiation.

**Figure 6:**
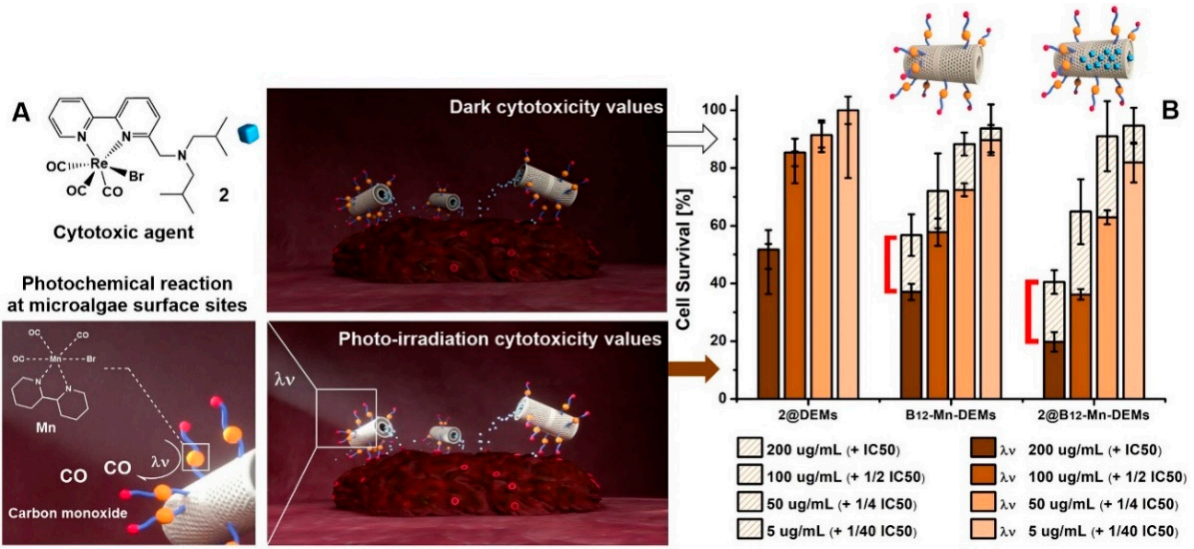
(**A**) Conceptual representation of the experiment, structure of complex **2** and detail of the photochemical reaction taking place at the microalgae surface sites (CO release from Mn complex). (**B**) Histograms representing the effects on HCT-116 colorectal cancer cells % survival by different doses of **2**@DEMs, B_12_-Mn-DEMs and **2**@B_12_-Mn-DEMs when administered in the dark (empty bars) and under the same conditions following light activation of the microalgae surface (filled colored bars). The red square brackets on the graph are meant to highlight the enhanced cytotoxicity of the material upon photo-irradiation.

The cytotoxicity of the **1**@B_12_-ZnTCPP-DEMs and **2**@B_12_-Mn-DEMs formulations in dark and under light irradiation were assessed using the human colorectal HCT-116 cell line (Figure 5 and Figure 6). In the case of **1**@B_12_-ZnTCPP-DEMs, light activation promotes singlet oxygen generation by the ZnTCPP component. Photoactivation of **1**@B_12_-ZnTCPP-DEMs increased the cytotoxicity of the construct up to 2.4-fold for the most concentrated sample (λν 200 μg/mL (+IC_50_), Figure 5 and Table 2) when compared to the effects of the administration of **1** alone. Notably, **1**@B_12_-ZnTCPP-DEMs containing only 25% of the IC_50_ concentration value of **1**, caused a 45% increase in cell death upon light exposure in comparison to dark conditions, essentially reaching the cell-killing effects of the IC_50_ value of **1** at ¼ the complex concentration. **1**@B_12_-ZnTCPP-DEMs with 2.5% the IC_50_ of **1** caused an increase of 14% of cell death under tested similar conditions. Overall, as it can be appreciated from the graphs in Figure 5 that the data suggest that lower amounts of cytotoxic compounds are needed in combination with the photoactivatable surface-functionalized diatom microalgae for an equally effective drug formulation or cytotoxic effect. Unmodified natural DEMs, either in the dark or exposed to light, showed no toxicity at concentrations >100 µg/mL, while both DEMs containing adsorbed **1** and B_12_-TCPP-DEMs particles alone showed slightly higher activity upon the light exposure (Figure 5). The photoactivity of complex **1** was somewhat surprising. We posit that it may be due to activation of the pending α-diimine ligand-CH_2_-Br arm towards nucleophilic substitution with bio-available amines via reactions of the complexes previously [48]. 

For **2**@B_12_-Mn-DEMs, light activation induces CO release from the photoCORM Mn component at the surface of the microalgae. CO released from this type of molecule is known to promote pro-apoptotic effects in different cancer cell lines [85,86,87,88,89,90] via CO-mediated attenuation of glutathione and nuclear metallothionein levels [91] and inhibition of cystathionine β-synthase [92]. When HCT-116 cells were exposed to **2**@B_12_-Mn-DEMs, photoactivation of the material similarly increased the overall cytotoxicity effect of complex **2**. Light irradiation of **2**@B_12_-Mn-DEMs increased the cytotoxicity of the construct by ca. 2.0-fold in the case of the most concentrated sample (λν 200 μg/mL (+IC_50_), Figure 6 and Table 2) when compared to the same sample in the dark or to the effects of the administration of **2** alone. In particular, **2**@B_12_-Mn-DEMs containing 25% of the IC_50_ concentration value of **2** caused a 31% increase in cell death upon light exposure in comparison to dark conditions. Photoactivation of **2**@B_12_-Mn-DEMs containing ½ the IC_50_ concentration value of **2** was more effective than administration of **2** alone at its IC_50_ concentration or B_12_-Mn-DEMs, inducing ca. 64% cell death. Photoactivation of **2**@B_12_-Mn-DEMs containing only 2.5% of the same IC_50_ was not effective but still induced a 13% increase of cell death compared to the same concentrations of **2** or B_12_-Mn-DEMs. As concluded for **1**@B_12_-ZnTCPP-DEMs, the data for **2**@B_12_-Mn-DEMs also suggest a potential of this poly-systemic approach in cancer treatment.

**Figure 7:**
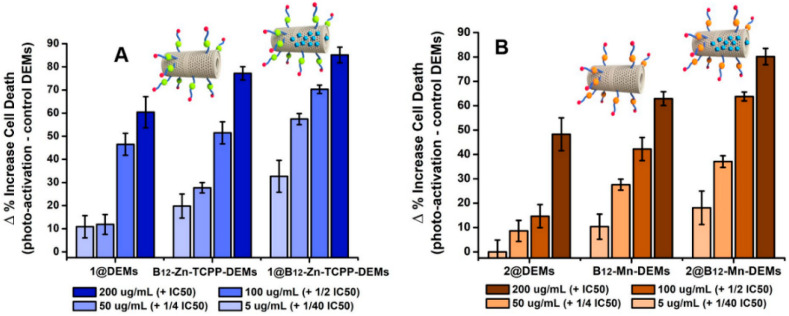
(**A**,**B**) Histograms representing the effect of light activation on different doses of 1@B_12_-ZnTCPP-DEMs and 2@B_12_-Mn-DEMs, respectively, on HCT-116 colorectal cancer cells.

Finally, by plotting the delta percentage of cell death measured upon light irradiation of raw DEMs, B_12_-ZnTCPP-DEMs, B_12_-Mn-DEMs and their respective combinations with rhenium anticancer complexes **1** and **2** (Figure 7) clearly demonstrate the improved efficiency of this approach (data not shown for raw DEMs alone, which are non-toxic). Data confirm the additive (and possible synergistic) effect of drugs **1** and **2** together with the ^1^O_2_-generating porphyrin and the CO-releasing photoCORM-functionalized microalgae surface, respectively.

## 4. Conclusions

In this study, we have reported the synthesis and characterization of two bio-inspired hybrid multifunctional drug delivery systems based on diatom microalgae. Both photoactivatable materials, either bearing a tetracarboxyphenyl porphyrin PS or tricarbonyl bipyridyl Mn(I) photo-CORM, are able to bind to HCT-116 colorectal cancer cells via the outer cobalamin layer and slowly release highly active rhenium(I) tricarbonyl anticancer complexes. The ability of the B_12_-*X*-DEMs micro-particles to retain a high level of surface functionalization in simulated gastric fluids was also demonstrated. Furthermore, the potentiation effect of the chemotoxic drug by the different light-activated molecules at the surface of diatom microalgae was demonstrated by MTT assay under dark/light conditions on the HCT-116 cell line. At least a 2-fold increase of cytotoxicity toward HCT-116 was observed upon light irradiaton for both constructs (at 200 μg mL^−1^, incubated with drug concentration corresponding to respective IC_50_ values) with cell survival falling from ca. 40% to ca. 20%. Our results are in line with what was reported earlier in the literature for different constructs [93,94,95,96,97], indicating that synergistic chemo-photodynamic therapy mediated by diatom microalgae is a strategy worth pursuing in colon-focused drug delivery. It is well known that undesirable side effects of most chemotherapeutics like cisplatin are dose-dependent [98]; thus, this approach can reduce the need for chemotherapeutics concentration for equal effectiveness, hence leading to diminished undesirable side effects for the patient. Future efforts for the optimization of this hybrid multifunctional drug delivery system will be directed towards coating formulations (e.g., pH-dependent polymers such as cellulose acetate phthalates, hydroxypropyl methyl-cellulose phthalate or biodegradable liposomes) that can respond to dynamic pH changes of the gastro-intestinal (GI) tract in order to expose the active material in the colon only. This approach should further increase surface coating retention after the passage through the stomach in future formulations.

## Figures and Tables

**Table 1:** Fluorescence quantum yield ($\Phi_{fl}$), lifetime (τf) and singlet oxygen quantum yield ($\Phi_{\Delta }$) of the porphyrin complexes in DMSO. Photo-induced CO-release kinetics of the manganese complexes in PBS buffer pH 7.4.

**Molecule**	$\Phi_{fl}$	**τ_f_ ± 0.1 [ns]**	$\Phi_{\Delta }$
ZnTCPP	0.009	4.7	0.77 ± 0.01
PEG-ZnTCPP	0.046	8.6	0.66 ± 0.10
B_12_-ZnTCPP	0.035	5.7	0.90 ± 0.03
	**t_1/2_ [s] ^a^**	**Equivalent of CO released ^b^**	
Mn ^c^	1.6 ± 1.1	1.3 ± 0.1	
B_12_-Mn	2.6 ± 1.4	1.3 ± 0.1	

^a^ Pseudo half-life, determined from UV/Vis spectral studies. ^b^ Determined via myoglobin assay. ^c^ (2,2′-bipyridine)-3,4′-dicarboxylic acid manganese tricarbonyl complex.

**Table 2:** Loading degrees of rhenium complexes **1** and **2** in % wt into DEMs and in vitro cytotoxicity (HCT-116 cells, IC_50_, µM) of complexes and when administered free, in DEMs in the dark (@B_12_-*X*-DEMs) and in DEMs following photoactivation of their surface (@B_12_-*X*-DEMs-λν).

Compound	µg drug/mg DEMs	IC_50_, µM
**1**		5.0 ± 0.1
**1**@B_12_-ZnTCPP-DEMs	38.9 (3.7%)	4.7 ± 0.1
**1**@B_12_-ZnTCPP-DEMs-λν	38.9 (3.7%)	1.2 ± 0.1
**2**		6.2 ± 0.2
**2**@B_12_-Mn-DEMs	15.9 (1.6%)	6.0 ± 0.2
**2**@B_12_-Mn-DEMs-λν	15.9 (1.6%)	3.1 ± 0.1

## Supplementary Materials

The following are available online at https://www.mdpi.com/1999-4923/12/5/480/s1: Figure S1: ^1^H-NMR of TCPP; Figure S2: ^1^H-NMR of B_12_-TCPP; Figure S3: ^1^H-NMR of TCPP-1; Figure S4: ^1^H-NMR of B_12_-Mn; Figure S5: MS spectrum of B_12_-TCPP; Figure S6: MS spectrum of TCPP-1; Figure S7: Fluorescence spectra of PEG-ZnTCPP and B_12_-ZnTCPP; Figure S8: Fluorescence lifetime decay curves of B_12_-ZnTCPP and ZnTCPP-1; Figure S9: IR of Mn; Figure S10: IR of B_12_-Mn; Figure S11: HPLC chromatogram of B_12_-TCPP; Figure S12: UV/Vis of Mn; Figure S13: UV/Vis of B_12_-Mn; Figure S14: Solid state UV/Vis of DEMs, Mn-DEMs, B_12_-DEMs and B_12_-Mn-DEMs; Figure S15: Solid state UV/Vis of DEMs, B_12_-TCPP-DEMs and B_12_-ZnTCPP-DEMs; Figure S16: Concentration of Mb-CO upon light irradiation of Mn; Figure S17: Concentration of Mb-CO upon light irradiation of B_12_-Mn; Figure S18: Spectral changes at 410 nm of Mn upon light irradiation; Figure S19: Spectral changes at 410 nm of B_12_-Mn upon light irradiation; Figure S20: Mn absorption decrease at 410 nm regarding irradiation time; Figure S21: B_12_-Mn absorption decrease at 365 nm regarding irradiation time; Figure S22: Calculated IR spectrum of B_12_-Mn (gas phase b3lyp/lanl2dz); Table S1: List of calculated IR frequencies for B_12_-Mn (gas phase b3lyp/lanl2dz); Figure S23: Calculated IR spectrum of B_12_-TCPP (gas phase b3lyp/lanl2dz); Table S2: List of calculated IR frequencies for B_12_-TCPP (gas phase b3lyp/lanl2dz).
