# Supplementary material for: Photoactivatable Surface-Functionalized Diatom Microalgae for Colorectal Cancer Targeted Delivery and Enhanced Cytotoxicity of Anticancer Complexes
Source: Pharmaceutics. 2020 May 25;12(5):480. doi: 10.3390/pharmaceutics12050480 (PMC7285135; doi:10.3390/pharmaceutics12050480)
Supplement: Supplementary file 1 [file pharmaceutics-12-00480-s001.pdf]

pharmaceutics

MDPI

Supplementary Materials: Photoactivatable Surface-Functionalized Diatom Microalgae for Colorectal Cancer Targeted Delivery and Enhanced Cytotoxicity of Anticancer Complexes

Joachim Delasoie, Philippe Schiel, Sandra Vojnovic and Jasmina Nikodinovic-Runic and Fabio Zobi *

NMR Spectra

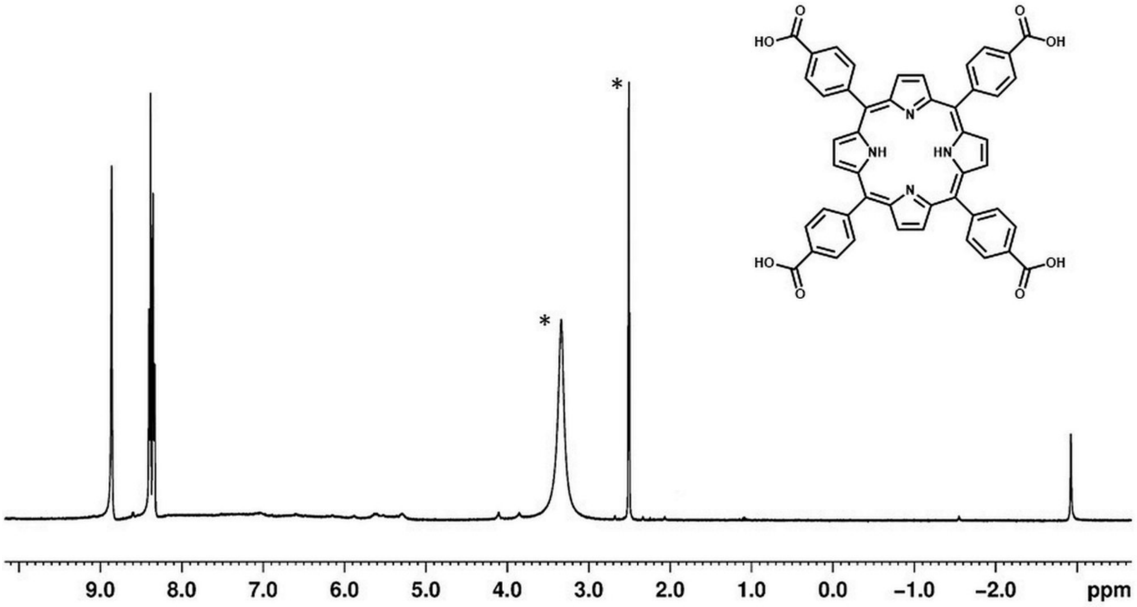

Figure S1. ¹H-NMR of TCPP. 400 MHz NMR in DMSO-d6 (✱= solvent signal).

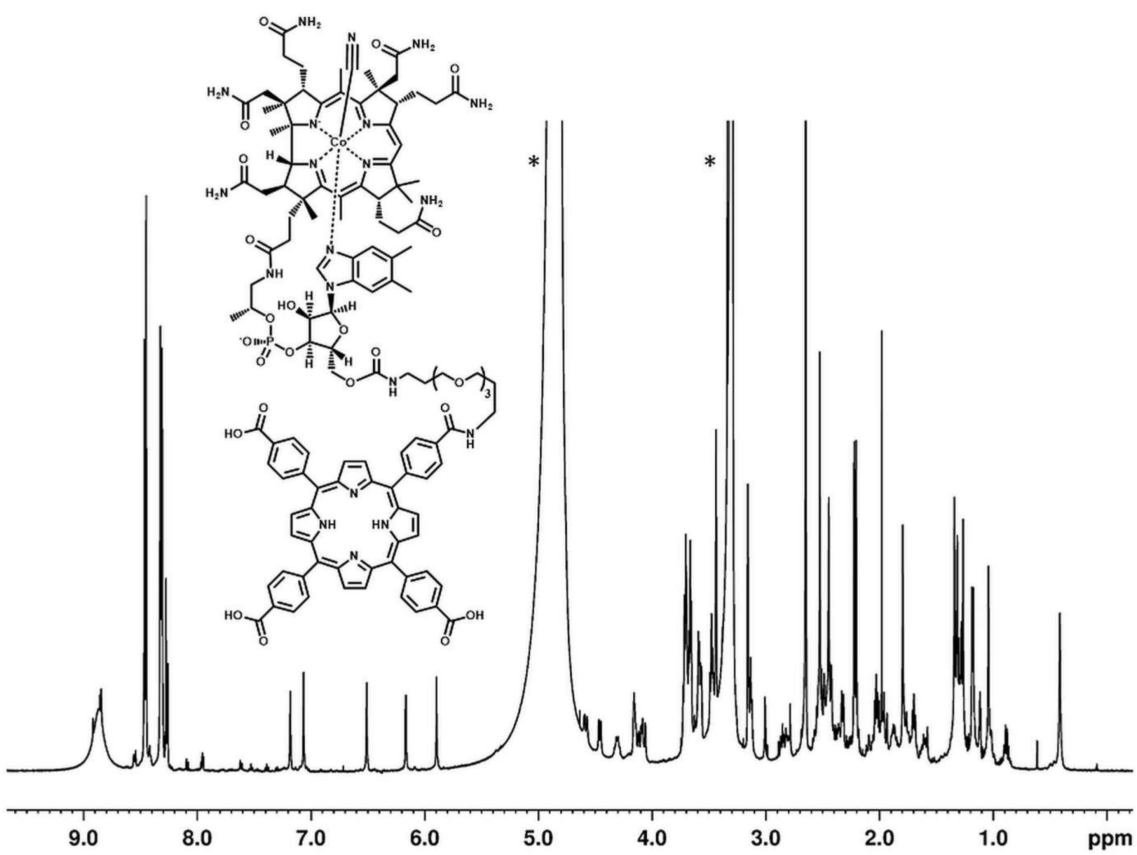

Figure S2. ^{1}H-NMR of B$_{12}$-TCPP. 500 MHz NMR in MeOD-d4 (✱= solvent signal).

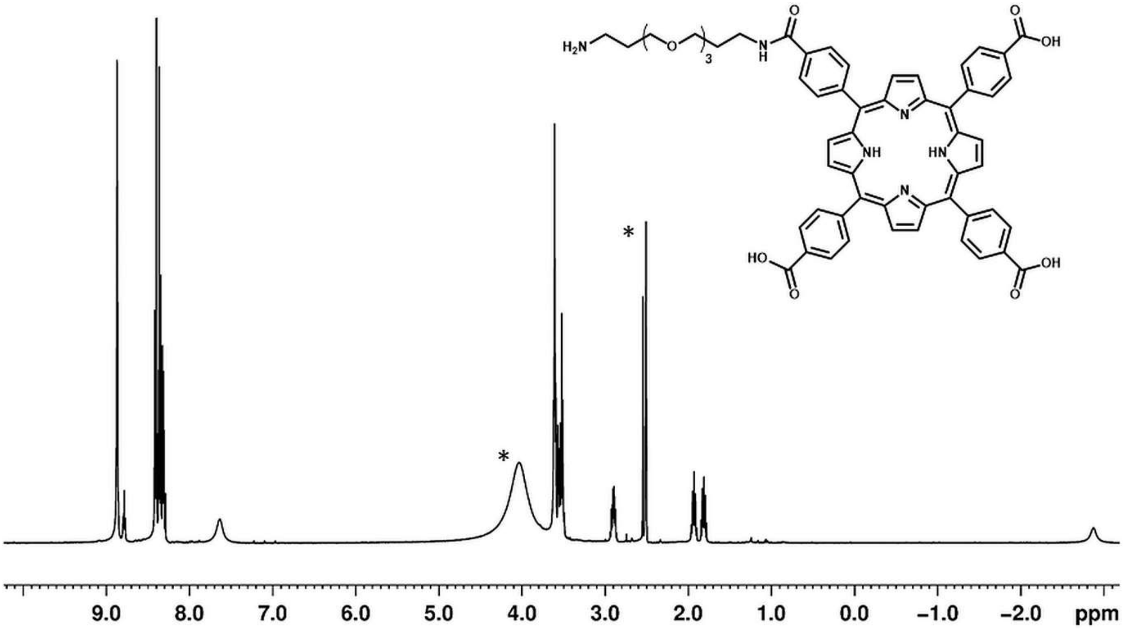

Figure S3. ^{1}H-NMR of TCPP-1. 400 MHz NMR in DMSO-d6 (✱= solvent signal).

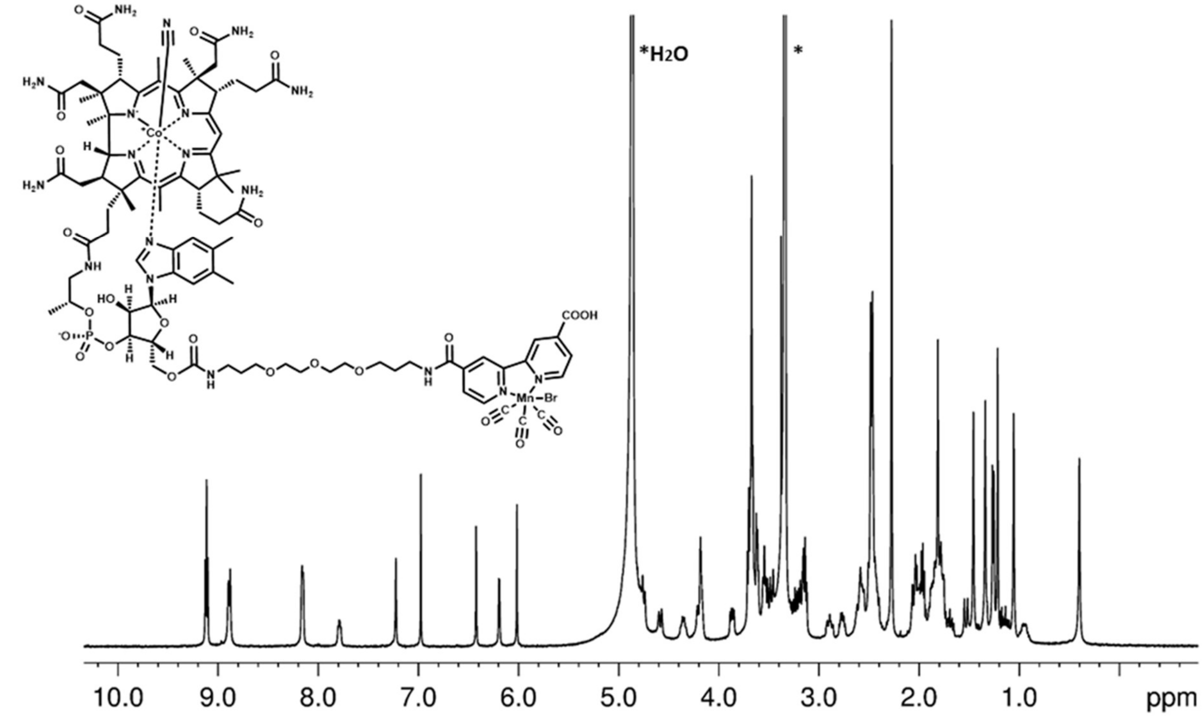

Figure S4. ^{1}H-NMR of B_{12}-Mn. 400 MHz NMR in MeOD-d4 (✱= solvent signal).

Mass Spectrometry

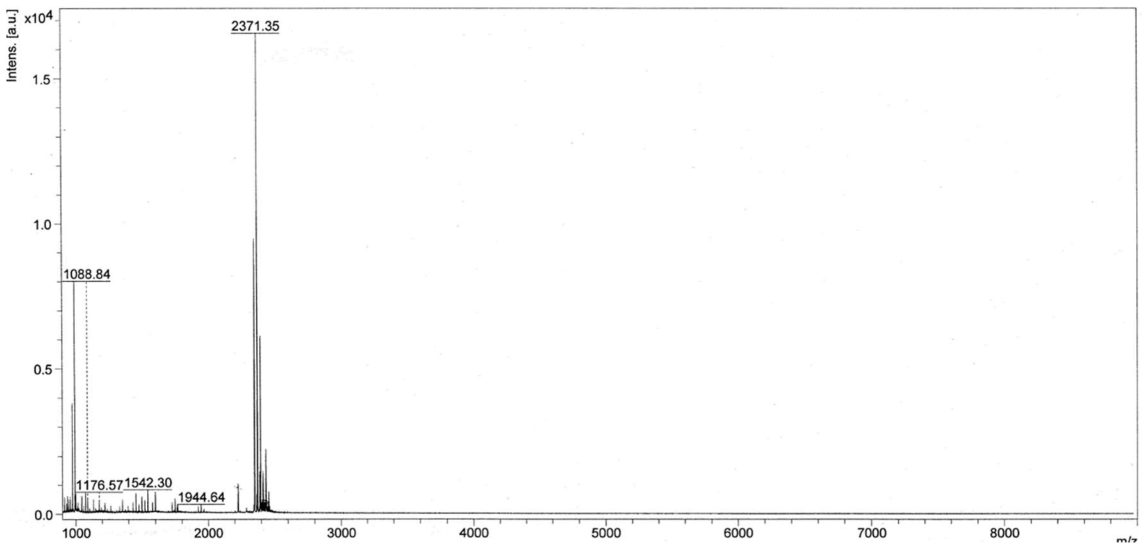

Figure S5. MS spectrum of B_{12}-TCPP. Measured with MALDI-TOF, 2,5-dihydroxybenzoic acid (DHB) used as matrix.

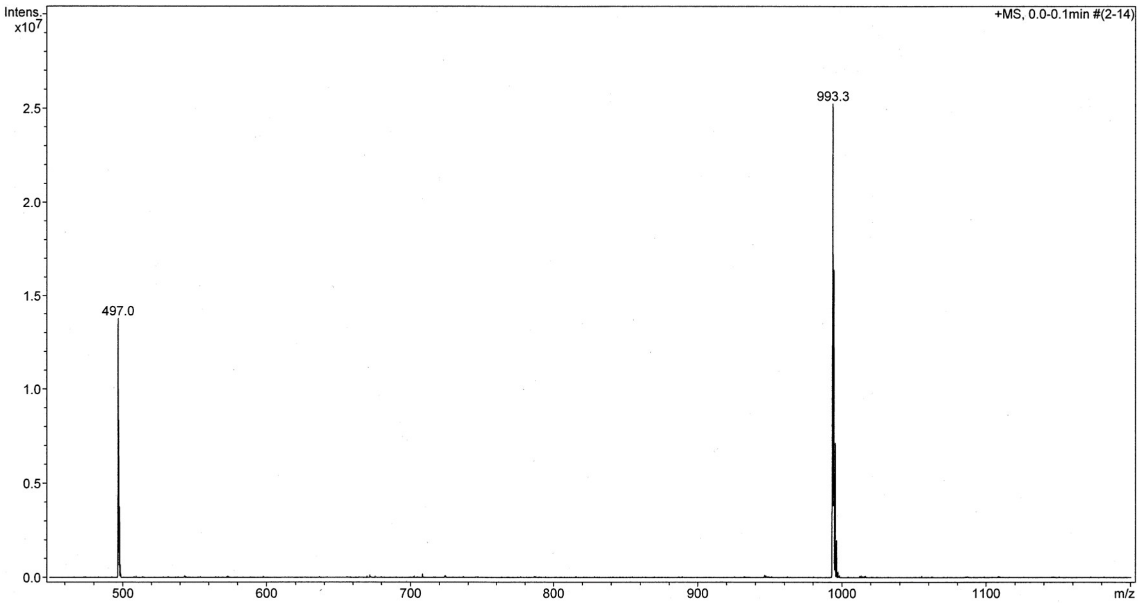

Figure S6. MS spectrum of TCPP-1. Measured with.ESI-MS (positive mode) in MeOH.

Fluorescence Spectroscopy

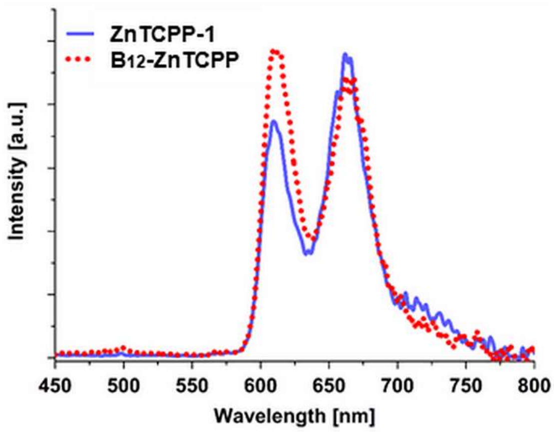

Figure S7. Fluorescence spectra of ZnTCPP-1 and B_{12}-ZnTCPP. Measured in DMSO.

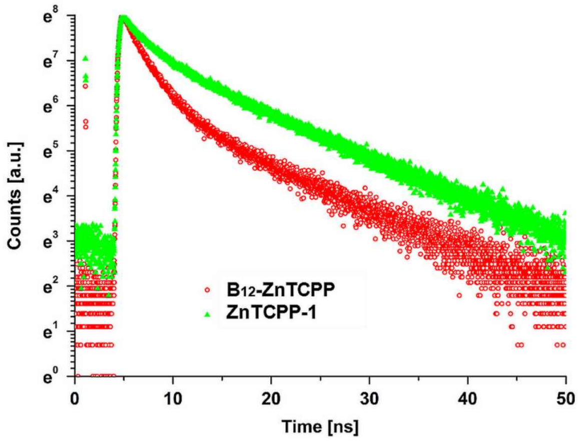

Figure S8. Fluorescence lifetime decay curves of B_{12}-ZnTCPP and ZnTCPP-1. The samples were prepared in DMSO and measurements were recorded with a TCSPC spectrophotometer.

Infrared Spectroscopy

All the IR spectra were measured with ATR system.

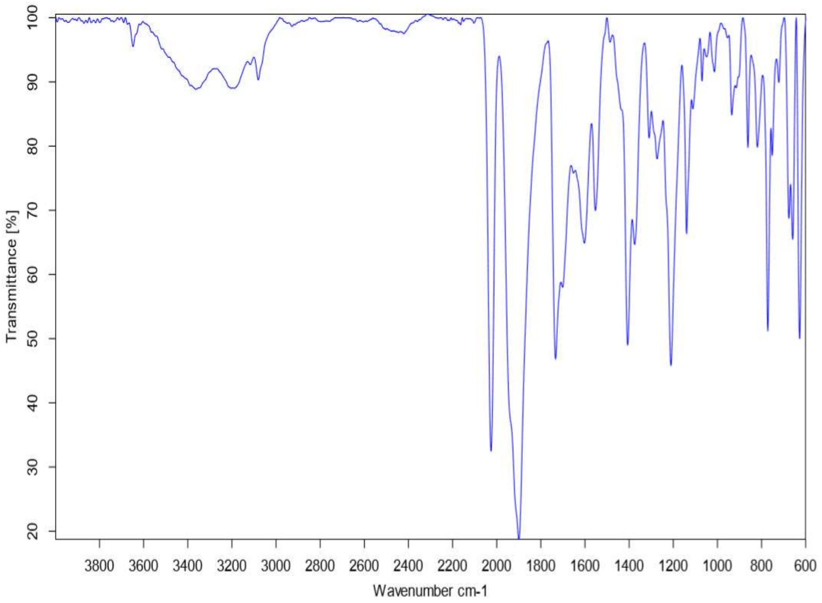

Figure S9. IR of Mn.

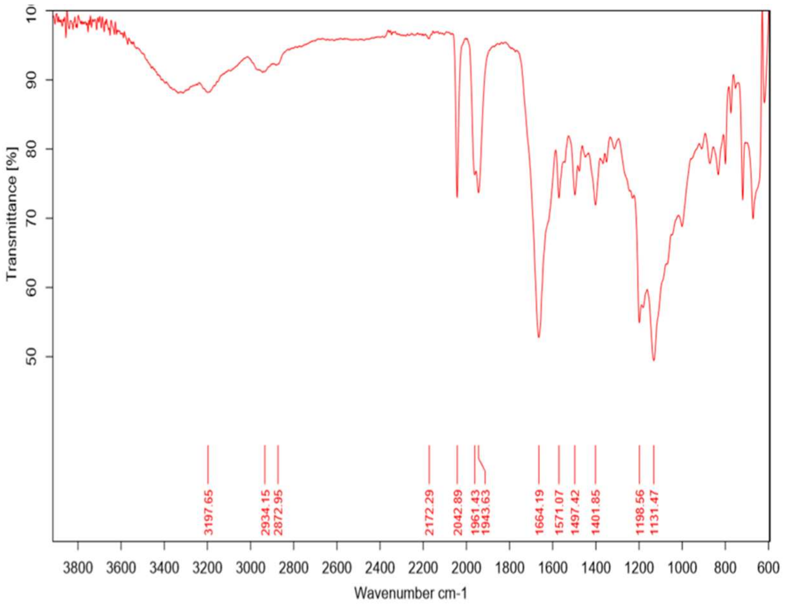

Figure S10. IR of B_{12}-Mn.

Spectra of Mn correspond to previously described analysis in literature[1].

[1] A. Ruggi and F. Zobi, "Quantum-CORMs: Quantum Dot Sensitized CO Releasing Molecules," *Dalton Trans.* 44, no. 24 (2015): 10928–31, https://doi.org/10.1039/C5DT01681A; Jeremie Rossier et al., "Organometallic Cobalamin Anticancer Derivatives for Targeted Prodrug Delivery via Transcobalamin-Mediated Uptake," *Dalton Trans.* 46, no. 7 (2017): 2159–64, https://doi.org/10.1039/C6DT04443C.

HPLC Analyse

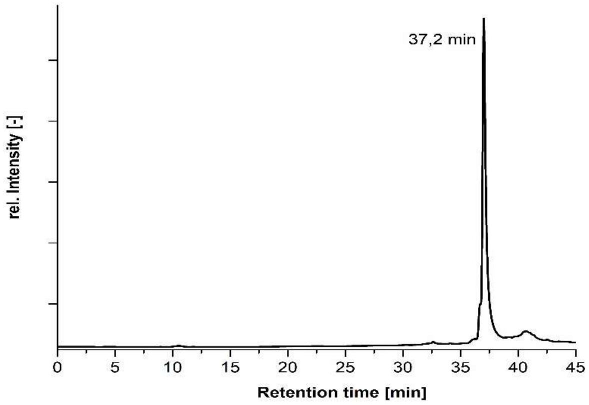

Figure S11. HPLC chromatogram of B12-TCPP. For analyticyl HPLC a Macherey-Nagel Nucleodur C18 HTec (5 µm particle size, 110 Å pore size, 250 × 21 mm) was used. Aqueous trifluoroacetic acid 0.1% solution and pure methanol were respectively used as solvents (A) and (B). The purified compound was injected and following gradient was used: 0–5 min (50% A), 5–30 (50% A → 0% A), 30–45 min (100% B), the flow rate set to 5 mL min^{-1} and the compound detected at 320 nm. The retention time of the compound corresponds to 37.2 minutes.

UV/Vis Spectrometry

In solution

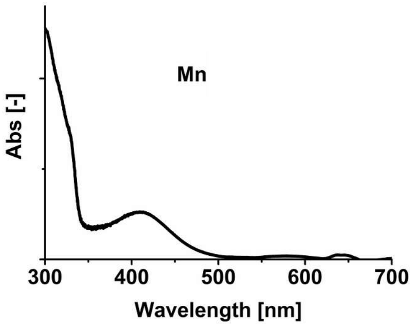

Figure S12. UV/Vis of Mn.

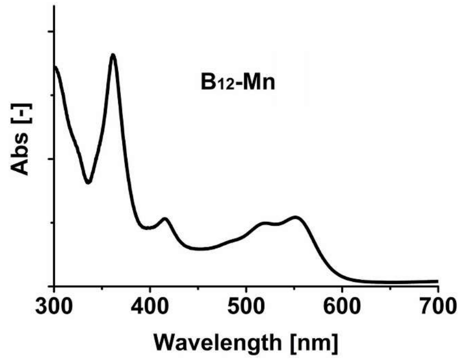

Figure S13. UV/Vis of B12-Mn.

In solid-state

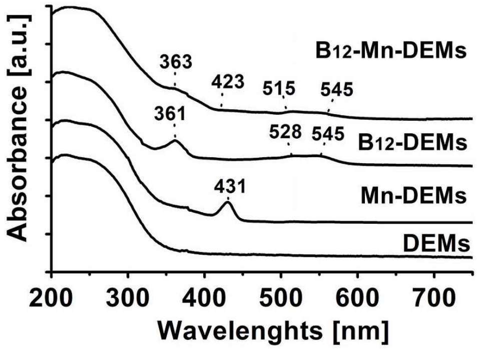

Figure S14. Solid state UV/Vis of DEMs, Mn-DEMs, B12-DEMs and B12-Mn-DEMs.

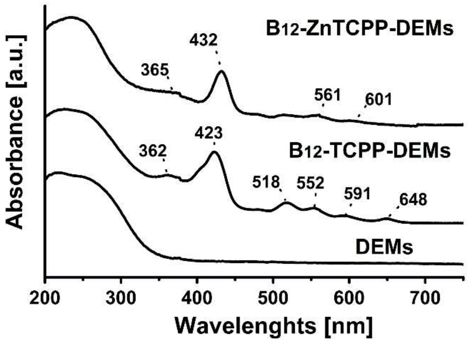

Figure S15. Solid state UV/Vis of DEMs, B12-TCPP-DEMs and B12-ZnTCPP-DEMs.

Equivalent of CO release

Here below are shown the concentration of Myoglobin-CO complex c(Mb-CO) formed through time with the CO release from either Mn5 (15 µM) or B12-Mn5 (10 µM) upon irradiation at 420 nm under

Argon atmosphere and determined from UV/Vis spectroscopy as described by Motterlini *et al* [2]. The solution were measured in 0.1M PBS buffer at pH 7.4, 10 mM dithionite, with respectively 45 and 30 μM Myoglobin for Mn and B_{12}-Mn assays. Due to its poor solubility, Mn was previously dissolved in DMSO (1% final concentration).

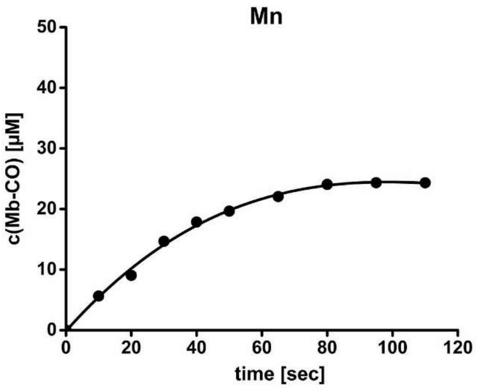

Figure S16. Concentration of Mb-CO upon light irradiation of Mn.

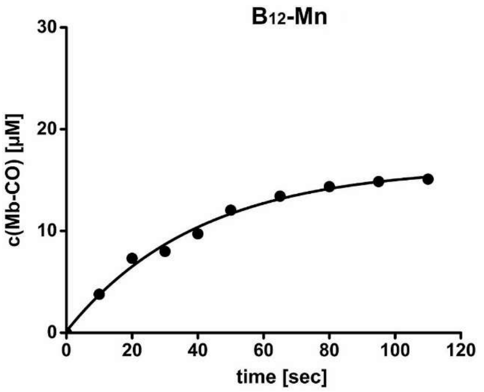

Figure S17. Concentration of Mb-CO upon light irradiation of B_{12}-Mn.

Half life

Monitoring of the spectral changes in the electronic absorption spectrum of compounds Mn and B_{12}-Mn in 0.1 M PBS upon irradiation with 420 nm light. The complexes Mn was previously dissolved in DMSO (1% final concentration).

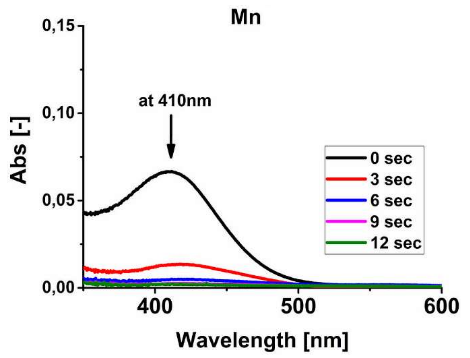

Figure S18. Spectral changes at 410 nm of Mn upon light irradiation.

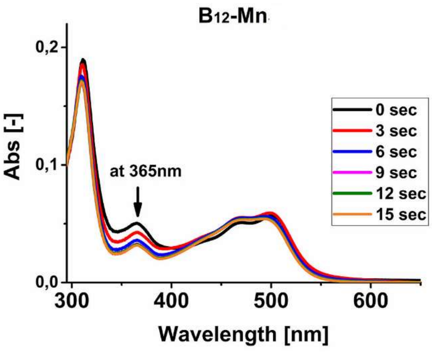

Figure S19. Spectral changes at 365 nm of B_{12}-Mn upon light irradiation.

[2] Roberto Motterlini and Leo E. Otterbein, "The Therapeutic Potential of Carbon Monoxide," *Nature Reviews Drug Discovery* 9 (September 1, 2010): 728.

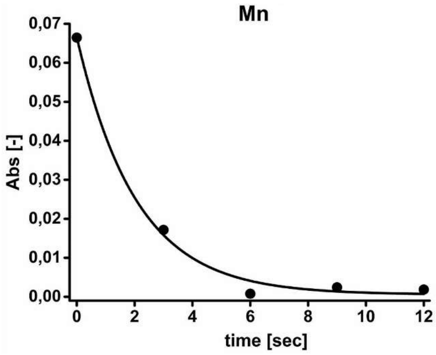

Figure S20. Mn absorption decrease at 410 nm regarding irradiation time.

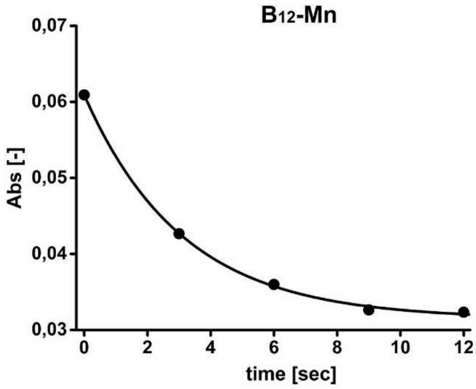

Figure S21. B_{12}-Mn absorption decrease at 365 nm regarding irradiation time.

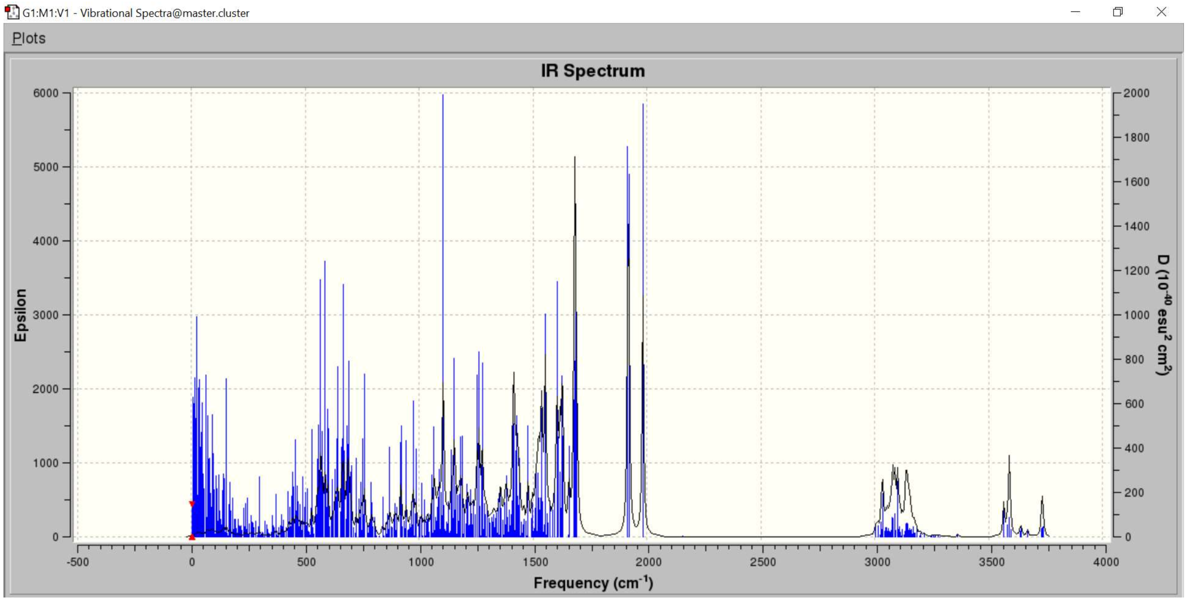

Figure S22. Calculate IR spectrum of B_{12}-Mn (gas phase b3lyp/lanl2dz).

Table S1. List of calculated IR frequencies for B_{12}-Mn (gas phase b3lyp/lanl2dz).

IR Spectrum	
X-Axis: Frequency	(cm^{-1})
Y-Axis: Epsilon	
X	Y
0.0000000000	8.8703694823
10.0000000000	27.3646439481
20.0000000000	52.1493719298
30.0000000000	75.6349061616
40.0000000000	63.8392044796
50.0000000000	71.3648041235
60.0000000000	58.5303282478

70.0000000000	99.7585892549
80.0000000000	97.4269779402
90.0000000000	112.6849065079
100.0000000000	92.9367247009
110.0000000000	68.8976208476
120.0000000000	70.0561646256
130.0000000000	64.6150408848
140.0000000000	124.0724562208
150.0000000000	126.6313021387
160.0000000000	68.7121825262
170.0000000000	81.4173881675
180.0000000000	56.8050446689

190.0000000000	47.7087173758
200.0000000000	28.0937743403
210.0000000000	29.1390839116
220.0000000000	29.3343755885
230.0000000000	57.4724829987
240.0000000000	59.6570918094
250.0000000000	38.0391230125
260.0000000000	50.9721140664
270.0000000000	50.9860892908
280.0000000000	43.8577926265
290.0000000000	46.6421515871
300.0000000000	64.5162456864
310.0000000000	25.9785862228
320.0000000000	33.3539825653
330.0000000000	39.9821438577
340.0000000000	29.6110444559
350.0000000000	38.9608016187
360.0000000000	47.8693392432
370.0000000000	88.4088372753
380.0000000000	36.3648976153
390.0000000000	54.5124317330
400.0000000000	73.5286373899
410.0000000000	55.7943127414
420.0000000000	89.0899442615
430.0000000000	206.9734909762
440.0000000000	213.6202597869
450.0000000000	195.4248546641
460.0000000000	273.4802554278
470.0000000000	225.2307725568
480.0000000000	151.7994004598
490.0000000000	204.5532306529
500.0000000000	170.8569377641
510.0000000000	190.6357080405
520.0000000000	166.7162985249
530.0000000000	327.3732258804
540.0000000000	196.6164631824
550.0000000000	509.9544471194
560.0000000000	625.3910556225
570.0000000000	973.2602225523
580.0000000000	562.2819191357
590.0000000000	666.8351022520
600.0000000000	662.6390962988
610.0000000000	233.6133759903
620.0000000000	318.8276075337
630.0000000000	560.5634858499
640.0000000000	633.6659660492

650.0000000000	396.0251763340
660.0000000000	845.5115562603
670.0000000000	790.5404516464
680.0000000000	671.4728920179
690.0000000000	985.7546629348
700.0000000000	545.7044991492
710.0000000000	190.2674211913
720.0000000000	241.2664937732
730.0000000000	177.1627364186
740.0000000000	338.6097784153
750.0000000000	505.0634366115
760.0000000000	646.0755111329
770.0000000000	182.4217308188
780.0000000000	180.3229101307
790.0000000000	308.5410493567
800.0000000000	195.0124799580
810.0000000000	82.9097330300
820.0000000000	40.8160232582
830.0000000000	51.4044796002
840.0000000000	163.5839087296
850.0000000000	117.0005979515
860.0000000000	220.1677221017
870.0000000000	375.2079527671
880.0000000000	187.1167977115
890.0000000000	274.1323876439
900.0000000000	301.1053026104
910.0000000000	225.7512583921
920.0000000000	709.7666583025
930.0000000000	235.0653593459
940.0000000000	340.4706779580
950.0000000000	213.9830261959
960.0000000000	254.4931127636
970.0000000000	498.5347948044
980.0000000000	390.8255857804
990.0000000000	233.4665012232
1000.0000000000	196.1143267409
1010.0000000000	314.1836786745
1020.0000000000	233.1904702530
1030.0000000000	240.9399425468
1040.0000000000	264.6812057847
1050.0000000000	322.1700328941
1060.0000000000	535.5208164037
1070.0000000000	621.2804200040
1080.0000000000	589.0285595310
1090.0000000000	696.8552421211
1100.0000000000	1153.5561585791

1110.0000000000	1056.6210992386
1120.0000000000	441.6977821547
1130.0000000000	380.2001790765
1140.0000000000	711.5121998795
1150.0000000000	1019.3309338670
1160.0000000000	883.9449953854
1170.0000000000	658.4859336146
1180.0000000000	803.2285797052
1190.0000000000	542.7755433517
1200.0000000000	324.8291004133
1210.0000000000	552.2701818007
1220.0000000000	431.2073852446
1230.0000000000	448.2778282560
1240.0000000000	430.7651568282
1250.0000000000	770.0186310642
1260.0000000000	1277.9095191709
1270.0000000000	841.4837326156
1280.0000000000	914.9990715080
1290.0000000000	508.5936968939
1300.0000000000	348.8060461602
1310.0000000000	303.8631146491
1320.0000000000	328.4926800468
1330.0000000000	372.1020036448
1340.0000000000	361.2799826889
1350.0000000000	498.7014659574
1360.0000000000	593.6835431215
1370.0000000000	429.3407275503
1380.0000000000	721.9683671366
1390.0000000000	511.8188644045
1400.0000000000	548.9449118171
1410.0000000000	1486.4405201879
1420.0000000000	1635.5784658767
1430.0000000000	1528.5432555738
1440.0000000000	897.6232330655
1450.0000000000	383.2850089842
1460.0000000000	439.1445346916
1470.0000000000	372.1599773571
1480.0000000000	703.9359876725
1490.0000000000	353.1687639225
1500.0000000000	584.2912543840
1510.0000000000	806.0103976103
1520.0000000000	1209.5751397191
1530.0000000000	1309.2860291591
1540.0000000000	1734.8638874523
1550.0000000000	1754.5256652119
1560.0000000000	973.5342216828

1570.0000000000	489.9063032969
1580.0000000000	341.5895608381
1590.0000000000	470.9044465137
1600.0000000000	1116.2505983634
1610.0000000000	1550.1943546138
1620.0000000000	1746.6626672089
1630.0000000000	2121.8165854868
1640.0000000000	437.0218912136
1650.0000000000	380.4154090185
1660.0000000000	699.5579668298
1670.0000000000	681.7645477789
1680.0000000000	3374.3077109920
1690.0000000000	2475.8937006849
1700.0000000000	605.4491159782
1710.0000000000	203.7479236636
1720.0000000000	105.0770207935
1730.0000000000	65.5036222063
1740.0000000000	45.1688186095
1750.0000000000	33.2238190705
1760.0000000000	27.3707526957
1770.0000000000	23.0705107139
1780.0000000000	19.4458816932
1790.0000000000	16.6952297117
1800.0000000000	15.4163853026
1810.0000000000	15.2995999748
1820.0000000000	15.4772442567
1830.0000000000	19.2084631610
1840.0000000000	21.4671743005
1850.0000000000	23.0450916781
1860.0000000000	30.1557895992
1870.0000000000	42.9731054309
1880.0000000000	66.7230295148
1890.0000000000	119.1452941722
1900.0000000000	277.3567851392
1910.0000000000	1239.8479077438
1920.0000000000	4120.8448197696
1930.0000000000	663.3885312910
1940.0000000000	220.6085303387
1950.0000000000	140.5212367656
1960.0000000000	157.2915459699
1970.0000000000	359.8905484507
1980.0000000000	2607.9862882497
1990.0000000000	710.7963758627
2000.0000000000	173.9631424392
2010.0000000000	78.4917380417
2020.0000000000	45.7379345017

2030.0000000000	30.4834625438	2490.0000000000	0.0000000000
2040.0000000000	22.0346382975	2500.0000000000	0.0000000000
2050.0000000000	16.8082809701	2510.0000000000	0.0000000000
2060.0000000000	13.3200753530	2520.0000000000	0.0000000000
2070.0000000000	10.8602609761	2530.0000000000	0.0000000000
2080.0000000000	7.3180164186	2540.0000000000	0.0000000000
2090.0000000000	4.6225204674	2550.0000000000	0.0000000000
2100.0000000000	3.8841731986	2560.0000000000	0.0000000000
2110.0000000000	3.3198489848	2570.0000000000	0.0000000000
2120.0000000000	2.8897701641	2580.0000000000	0.0000000000
2130.0000000000	2.5843322152	2590.0000000000	0.0000000000
2140.0000000000	2.4793211807	2600.0000000000	0.0000000000
2150.0000000000	1.5532344019	2610.0000000000	0.0000000000
2160.0000000000	4.0154749903	2620.0000000000	0.0000000000
2170.0000000000	0.5433081231	2630.0000000000	0.0000000000
2180.0000000000	0.1845550730	2640.0000000000	0.0000000000
2190.0000000000	0.0910529682	2650.0000000000	0.0000000000
2200.0000000000	0.0539574531	2660.0000000000	0.0000000000
2210.0000000000	0.0356252934	2670.0000000000	0.0000000000
2220.0000000000	0.0252569333	2680.0000000000	0.0000000000
2230.0000000000	0.0188314859	2690.0000000000	0.0000000000
2240.0000000000	0.0145776122	2700.0000000000	0.0000000000
2250.0000000000	0.0116170400	2710.0000000000	0.0000000000
2260.0000000000	0.0094742838	2720.0000000000	0.0000000000
2270.0000000000	0.0078737910	2730.0000000000	0.0000000000
2280.0000000000	0.0066469609	2740.0000000000	0.0000000000
2290.0000000000	0.0056859506	2750.0000000000	0.0000000000
2300.0000000000	0.0049191828	2760.0000000000	0.0000000000
2310.0000000000	0.0042976403	2770.0000000000	0.0000000000
2320.0000000000	0.0000000000	2780.0000000000	0.0000000000
2330.0000000000	0.0000000000	2790.0000000000	0.0000000000
2340.0000000000	0.0000000000	2800.0000000000	0.0000000000
2350.0000000000	0.0000000000	2810.0000000000	0.0000000000
2360.0000000000	0.0000000000	2820.0000000000	0.0000000000
2370.0000000000	0.0000000000	2830.0000000000	0.0000000000
2380.0000000000	0.0000000000	2840.0000000000	0.0000000000
2390.0000000000	0.0000000000	2850.0000000000	0.1011244620
2400.0000000000	0.0000000000	2860.0000000000	0.2057402378
2410.0000000000	0.0000000000	2870.0000000000	0.4214722512
2420.0000000000	0.0000000000	2880.0000000000	0.9760860702
2430.0000000000	0.0000000000	2890.0000000000	1.2574898587
2440.0000000000	0.0000000000	2900.0000000000	1.6848284663
2450.0000000000	0.0000000000	2910.0000000000	2.1484420936
2460.0000000000	0.0000000000	2920.0000000000	2.9441885772
2470.0000000000	0.0000000000	2930.0000000000	4.0930319127
2480.0000000000	0.0000000000	2940.0000000000	5.2255814008

2950.0000000000	7.0196407838
2960.0000000000	9.1114950982
2970.0000000000	12.2421844916
2980.0000000000	18.0581025876
2990.0000000000	30.9925153846
3000.0000000000	90.4704818774
3010.0000000000	180.7040717208
3020.0000000000	184.6122905284
3030.0000000000	598.8004440218
3040.0000000000	461.4423274165
3050.0000000000	349.7316061815
3060.0000000000	385.7465084897
3070.0000000000	535.6892215809
3080.0000000000	981.3492644286
3090.0000000000	925.8301787668
3100.0000000000	895.8393512523
3110.0000000000	476.5955534432
3120.0000000000	345.7473756100
3130.0000000000	395.3895003119
3140.0000000000	924.4269673751
3150.0000000000	637.5172714860
3160.0000000000	412.7081333970
3170.0000000000	264.8813721159
3180.0000000000	160.0473953157
3190.0000000000	77.9647539137
3200.0000000000	61.7954168491
3210.0000000000	40.1057623168
3220.0000000000	39.4153981027
3230.0000000000	15.5380133050
3240.0000000000	15.6455944314
3250.0000000000	29.8586568177
3260.0000000000	24.9122881607
3270.0000000000	16.2922297830
3280.0000000000	14.0496798851
3290.0000000000	5.2191733035
3300.0000000000	2.6072667406
3310.0000000000	1.5937902393
3320.0000000000	1.1072561544
3330.0000000000	1.0444659341
3340.0000000000	1.2580486640
3350.0000000000	2.6207815327
3360.0000000000	13.2796066289
3370.0000000000	18.1976997344
3380.0000000000	2.9177363894
3390.0000000000	1.0996957025
3400.0000000000	0.5824364192

3410.0000000000	0.6351622934
3420.0000000000	0.5543439930
3430.0000000000	0.6464214652
3440.0000000000	1.5085691864
3450.0000000000	1.7011625463
3460.0000000000	1.9591045076
3470.0000000000	2.2999964427
3480.0000000000	2.8031817480
3490.0000000000	3.5080782108
3500.0000000000	4.4040593135
3510.0000000000	5.7214532486
3520.0000000000	7.8525988645
3530.0000000000	11.3799272190
3540.0000000000	18.4464979817
3550.0000000000	37.0388654448
3560.0000000000	132.9114123435
3570.0000000000	339.8063059867
3580.0000000000	326.0118688193
3590.0000000000	1044.8748415546
3600.0000000000	287.7505826696
3610.0000000000	68.5531883668
3620.0000000000	36.0641527324
3630.0000000000	40.8019631205
3640.0000000000	142.0984319597
3650.0000000000	50.8718155315
3660.0000000000	27.0089001432
3670.0000000000	99.8981814843
3680.0000000000	25.8203782414
3690.0000000000	13.4320401908
3700.0000000000	13.4419526585
3710.0000000000	19.7100788901
3720.0000000000	44.5893333203
3730.0000000000	237.0520080876
3740.0000000000	388.6444388066
3750.0000000000	59.2744713958
3760.0000000000	20.0530020174
3770.0000000000	10.0901673110
3780.0000000000	6.0960045482
3790.0000000000	4.0969656680
3800.0000000000	2.9058685037
3810.0000000000	2.1275934339
3820.0000000000	1.6579069363
3830.0000000000	1.3289712633
3840.0000000000	1.0343134868
3850.0000000000	0.8605382923
3860.0000000000	0.7271424908

3870.0000000000	0.6225201484
3880.0000000000	0.5389540704
3890.0000000000	0.4711509119
3900.0000000000	0.0000000000
3910.0000000000	0.0000000000
3920.0000000000	0.0000000000
3930.0000000000	0.0000000000

3940.0000000000	0.0000000000
3950.0000000000	0.0000000000
3960.0000000000	0.0000000000
3970.0000000000	0.0000000000
3980.0000000000	0.0000000000
3990.0000000000	0.0000000000

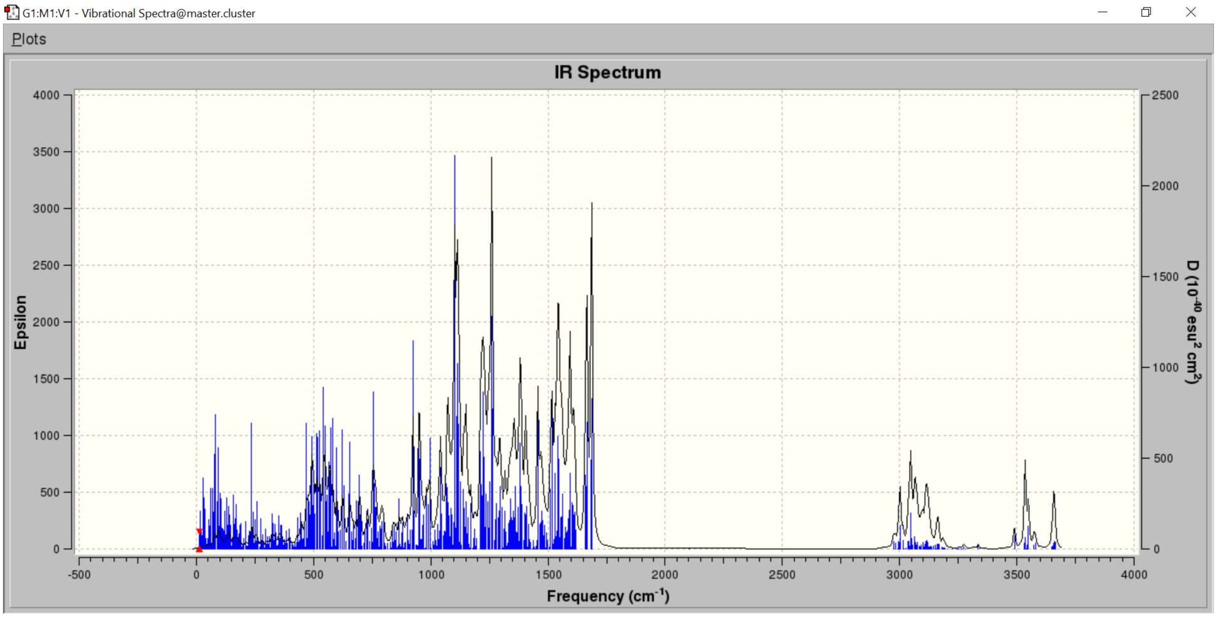

Figure S23. Calculate IR spectrum of B12-TCPP (gas phase b3lyp/lanl2dz).

Table S2. List of calculated IR frequencies for B12-TCPP (gas phase b3lyp/lanl2dz).

IR Spectrum	
X-Axis: Frequency	(cm^{-1})
Y-Axis: Epsilon	
X	Y
0.0000000000	2.6142602577
10.0000000000	5.8185099518
20.0000000000	14.2430170126
30.0000000000	26.5527897094
40.0000000000	23.1090105894
50.0000000000	27.5800716911
60.0000000000	46.4354538431
70.0000000000	59.9278385877
80.0000000000	153.9651727780
90.0000000000	128.5619690614
100.0000000000	129.1216152766
110.0000000000	79.2122268816
120.0000000000	72.4322755149
130.0000000000	95.6137853143
140.0000000000	149.4077435430
150.0000000000	69.3283884068
160.0000000000	92.9083030282
170.0000000000	109.6162394514
180.0000000000	68.5103089316
190.0000000000	58.6819536876
200.0000000000	40.0923993356
210.0000000000	61.7600937621
220.0000000000	53.1503854407

230.0000000000	99.4033919341
240.0000000000	171.0795681743
250.0000000000	115.5962626078
260.0000000000	103.5121174834
270.0000000000	46.5121498780
280.0000000000	73.9778934802
290.0000000000	44.9320957279
300.0000000000	64.7727275062
310.0000000000	61.7518339405
320.0000000000	117.3350900596
330.0000000000	127.5254172964
340.0000000000	80.3875584662
350.0000000000	112.1234154303
360.0000000000	129.5237127389
370.0000000000	77.2626559944
380.0000000000	69.9329683535
390.0000000000	65.2524224097
400.0000000000	86.8954873260
410.0000000000	51.6701385330
420.0000000000	56.1533984661
430.0000000000	120.4544936096
440.0000000000	139.4064358181
450.0000000000	241.1316928174
460.0000000000	207.5240066861
470.0000000000	472.7097748270
480.0000000000	471.8882790843
490.0000000000	692.7605171353

500.0000000000	702.9368158056
510.0000000000	552.5967375924
520.0000000000	493.7459451901
530.0000000000	532.5361820288
540.0000000000	697.9585142109
550.0000000000	825.4933755674
560.0000000000	529.5392735214
570.0000000000	724.2714592830
580.0000000000	488.9166771483
590.0000000000	315.1851667836
600.0000000000	433.5869357057
610.0000000000	189.1395709832
620.0000000000	367.3814529139
630.0000000000	341.8795990959
640.0000000000	144.4738803960
650.0000000000	318.6193778819
660.0000000000	306.8649582005
670.0000000000	238.6169293083
680.0000000000	278.4678157403
690.0000000000	287.3310665206
700.0000000000	426.9506017191
710.0000000000	130.5863659894
720.0000000000	177.0524656803
730.0000000000	354.2063959549
740.0000000000	208.0288381716
750.0000000000	547.3435252677
760.0000000000	617.9510740567
770.0000000000	348.5904170704
780.0000000000	236.0247490227
790.0000000000	366.0906562335
800.0000000000	285.6965010068
810.0000000000	89.0766131899
820.0000000000	63.2689928655
830.0000000000	110.9429831785
840.0000000000	235.4663353101
850.0000000000	218.3354270571
860.0000000000	175.9711104497
870.0000000000	223.5356540350
880.0000000000	281.6548129792
890.0000000000	201.8857491374
900.0000000000	306.1596787713
910.0000000000	270.7368603212
920.0000000000	736.6679630594
930.0000000000	500.1623704383
940.0000000000	313.2159084469
950.0000000000	1316.2797547522

960.0000000000	519.4986528079
970.0000000000	401.9629560312
980.0000000000	510.1918385849
990.0000000000	530.9565351924
1000.0000000000	358.7034395754
1010.0000000000	184.9342548066
1020.0000000000	274.8754480105
1030.0000000000	440.3591671507
1040.0000000000	998.1808956442
1050.0000000000	429.2081918206
1060.0000000000	454.5937119475
1070.0000000000	1209.8121582573
1080.0000000000	933.7847894852
1090.0000000000	866.2844334261
1100.0000000000	2540.7599417810
1110.0000000000	2486.2955386606
1120.0000000000	1932.0279856256
1130.0000000000	731.3195723856
1140.0000000000	1098.9405175303
1150.0000000000	1310.1107544356
1160.0000000000	599.2818513824
1170.0000000000	570.9575158560
1180.0000000000	276.2907063692
1190.0000000000	336.2035833266
1200.0000000000	305.1608381750
1210.0000000000	1181.6827102515
1220.0000000000	1845.0597599949
1230.0000000000	1517.8835123284
1240.0000000000	1130.9917445061
1250.0000000000	1632.3626367296
1260.0000000000	3512.7443090660
1270.0000000000	888.5940053427
1280.0000000000	795.7994560391
1290.0000000000	827.9036433715
1300.0000000000	698.1037387716
1310.0000000000	466.5025555234
1320.0000000000	571.7366376608
1330.0000000000	618.3042955483
1340.0000000000	834.6563806322
1350.0000000000	941.2297486977
1360.0000000000	995.8111476653
1370.0000000000	811.5176346028
1380.0000000000	1627.4329728115
1390.0000000000	972.6214464015
1400.0000000000	840.8098052614
1410.0000000000	799.0529007487

1420.0000000000	523.1002148235
1430.0000000000	212.7587857380
1440.0000000000	226.3196020397
1450.0000000000	523.0775759043
1460.0000000000	1001.2720293034
1470.0000000000	856.5524854544
1480.0000000000	559.7019457682
1490.0000000000	363.8007896753
1500.0000000000	345.2527631234
1510.0000000000	898.6173651667
1520.0000000000	1103.1565348518
1530.0000000000	1281.3929037367
1540.0000000000	1994.1379350543
1550.0000000000	1574.3870662322
1560.0000000000	1264.6254309025
1570.0000000000	754.5592948699
1580.0000000000	833.5714195506
1590.0000000000	1418.3896624546
1600.0000000000	1122.6207743231
1610.0000000000	1273.6524647955
1620.0000000000	544.6316353653
1630.0000000000	188.0584418743
1640.0000000000	172.4738981652
1650.0000000000	334.3946472056
1660.0000000000	1361.2425006163
1670.0000000000	1343.2835140677
1680.0000000000	1271.7109986331
1690.0000000000	2001.0362942331
1700.0000000000	320.2396472039
1710.0000000000	124.6196279263
1720.0000000000	67.9512149278
1730.0000000000	43.5057721133
1740.0000000000	30.4314892870
1750.0000000000	22.2983170799
1760.0000000000	16.3666592736
1770.0000000000	12.3194838970
1780.0000000000	9.6474404505
1790.0000000000	7.9837498511
1800.0000000000	6.7184116257
1810.0000000000	5.7331431950
1820.0000000000	4.5940409924
1830.0000000000	2.6495602646
1840.0000000000	2.3162654315
1850.0000000000	0.0000000000
1860.0000000000	0.0000000000
1870.0000000000	0.0000000000

1880.0000000000	0.0000000000
1890.0000000000	0.0000000000
1900.0000000000	0.0000000000
1910.0000000000	0.0000000000
1920.0000000000	0.0000000000
1930.0000000000	0.0000000000
1940.0000000000	0.0000000000
1950.0000000000	0.0002040707
1960.0000000000	0.0002339225
1970.0000000000	0.0002708355
1980.0000000000	0.0003172247
1990.0000000000	0.0003766332
2000.0000000000	0.0004544279
2010.0000000000	0.0005590535
2020.0000000000	0.0007044197
2030.0000000000	0.0009147612
2040.0000000000	0.0012353960
2050.0000000000	0.0017592162
2060.0000000000	0.0027018610
2070.0000000000	0.0046620490
2080.0000000000	0.0098441631
2090.0000000000	0.0318770816
2100.0000000000	0.2590442483
2110.0000000000	0.0524863546
2120.0000000000	0.0129959278
2130.0000000000	0.0056324011
2140.0000000000	0.0031167839
2150.0000000000	0.0019729886
2160.0000000000	0.0013595279
2170.0000000000	0.0009930921
2180.0000000000	0.0007569651
2190.0000000000	0.0005959915
2200.0000000000	0.0004813733
2210.0000000000	0.0003968875
2220.0000000000	0.0003328314
2230.0000000000	0.0002831141
2240.0000000000	0.0002437557
2250.0000000000	0.0002120669
2260.0000000000	0.0001861774
2270.0000000000	0.0000000000
2280.0000000000	0.0000000000
2290.0000000000	0.0000000000
2300.0000000000	0.0000000000
2310.0000000000	0.0000000000
2320.0000000000	0.0000000000
2330.0000000000	0.0000000000

2340.0000000000	0.0000000000
2350.0000000000	0.0000000000
2360.0000000000	0.0000000000
2370.0000000000	0.0000000000
2380.0000000000	0.0000000000
2390.0000000000	0.0000000000
2400.0000000000	0.0000000000
2410.0000000000	0.0000000000
2420.0000000000	0.0000000000
2430.0000000000	0.0000000000
2440.0000000000	0.0000000000
2450.0000000000	0.0000000000
2460.0000000000	0.0000000000
2470.0000000000	0.0000000000
2480.0000000000	0.0000000000
2490.0000000000	0.0000000000
2500.0000000000	0.0000000000
2510.0000000000	0.0000000000
2520.0000000000	0.0000000000
2530.0000000000	0.0000000000
2540.0000000000	0.0000000000
2550.0000000000	0.0000000000
2560.0000000000	0.0000000000
2570.0000000000	0.0000000000
2580.0000000000	0.0000000000
2590.0000000000	0.0000000000
2600.0000000000	0.0000000000
2610.0000000000	0.0000000000
2620.0000000000	0.0000000000
2630.0000000000	0.0000000000
2640.0000000000	0.0000000000
2650.0000000000	0.0000000000
2660.0000000000	0.0000000000
2670.0000000000	0.0000000000
2680.0000000000	0.0000000000
2690.0000000000	0.0000000000
2700.0000000000	0.0000000000
2710.0000000000	0.0000000000
2720.0000000000	0.0000000000
2730.0000000000	0.0000000000
2740.0000000000	0.0000000000
2750.0000000000	0.0000000000
2760.0000000000	0.0000000000
2770.0000000000	0.0000000000
2780.0000000000	0.0000000000
2790.0000000000	0.0000000000

2800.0000000000	0.0000000000
2810.0000000000	0.0000000000
2820.0000000000	0.0696807727
2830.0000000000	0.1394035791
2840.0000000000	0.2927345561
2850.0000000000	0.6670844613
2860.0000000000	0.8579116713
2870.0000000000	1.0120420562
2880.0000000000	1.3637019706
2890.0000000000	2.1784100281
2900.0000000000	2.7637367878
2910.0000000000	3.8052684384
2920.0000000000	4.9511175938
2930.0000000000	6.4172727922
2940.0000000000	8.8719596968
2950.0000000000	13.1525191743
2960.0000000000	24.1858523185
2970.0000000000	91.3675826036
2980.0000000000	140.7359606536
2990.0000000000	147.2463284576
3000.0000000000	501.7258438632
3010.0000000000	207.8657435823
3020.0000000000	123.4129221647
3030.0000000000	184.3896290759
3040.0000000000	536.7062270552
3050.0000000000	715.2529978853
3060.0000000000	465.6039254603
3070.0000000000	568.9715434223
3080.0000000000	313.1827689428
3090.0000000000	315.2364343457
3100.0000000000	350.1154333347
3110.0000000000	488.7981254031
3120.0000000000	511.9411042302
3130.0000000000	257.6197931787
3140.0000000000	113.4684741570
3150.0000000000	111.9092318999
3160.0000000000	264.1778539531
3170.0000000000	149.9135330743
3180.0000000000	81.5157090173
3190.0000000000	82.4343163679
3200.0000000000	28.5307849572
3210.0000000000	18.3011275569
3220.0000000000	11.8448573560
3230.0000000000	8.5677780483
3240.0000000000	9.6495422523
3250.0000000000	14.2680574097

3260.0000000000	8.9999102239
3270.0000000000	34.8915943896
3280.0000000000	16.1138418115
3290.0000000000	4.2958596517
3300.0000000000	2.3718763478
3310.0000000000	2.2132571894
3320.0000000000	3.6256835398
3330.0000000000	17.4638477452
3340.0000000000	16.7029608161
3350.0000000000	3.1585599976
3360.0000000000	1.3963151784
3370.0000000000	0.8896355056
3380.0000000000	1.2345084189
3390.0000000000	1.5338092585
3400.0000000000	1.6744184040
3410.0000000000	1.9001091410
3420.0000000000	2.3721964586
3430.0000000000	2.8907548677
3440.0000000000	3.6472750610
3450.0000000000	4.9168227632
3460.0000000000	7.2802603087
3470.0000000000	12.9586002443
3480.0000000000	37.0741720903
3490.0000000000	220.3593160983
3500.0000000000	42.3237582355
3510.0000000000	34.3550288422
3520.0000000000	68.5414309254
3530.0000000000	353.9400390284
3540.0000000000	435.7943153721
3550.0000000000	452.3161328950
3560.0000000000	87.6093045561
3570.0000000000	120.3452864815
3580.0000000000	130.6700731442
3590.0000000000	27.9403934279
3600.0000000000	15.2566892866
3610.0000000000	12.5222997177
3620.0000000000	13.6119193542

3630.0000000000	19.9362994795
3640.0000000000	45.4069835805
3650.0000000000	221.8008282781
3660.0000000000	508.1040421739
3670.0000000000	96.8267304242
3680.0000000000	27.4087363154
3690.0000000000	13.1700380244
3700.0000000000	7.4412870220
3710.0000000000	4.6694488202
3720.0000000000	3.3056706455
3730.0000000000	2.4682603586
3740.0000000000	1.7947484174
3750.0000000000	1.4247135921
3760.0000000000	1.1584258604
3770.0000000000	0.9604162394
3780.0000000000	0.8091797302
3790.0000000000	0.6910599028
3800.0000000000	0.5970454178
3810.0000000000	0.4964233230
3820.0000000000	0.0799248647
3830.0000000000	0.0000000000
3840.0000000000	0.0000000000
3850.0000000000	0.0000000000
3860.0000000000	0.0000000000
3870.0000000000	0.0000000000
3880.0000000000	0.0000000000
3890.0000000000	0.0000000000
3900.0000000000	0.0000000000
3910.0000000000	0.0000000000
3920.0000000000	0.0000000000
3930.0000000000	0.0000000000
3940.0000000000	0.0000000000
3950.0000000000	0.0000000000
3960.0000000000	0.0000000000
3970.0000000000	0.0000000000
3980.0000000000	0.0000000000
3990.0000000000	0.0000000000

Motterlini, Roberto, and Leo E. Otterbein. "The Therapeutic Potential of Carbon Monoxide." *Nature Reviews Drug Discovery* 9 (September 1, 2010): 728.

Rossier, Jeremie, Daniel Hauser, Emmanuel Kottelat, Barbara Rothen-Rutishauser, and Fabio Zobi. "Organometallic Cobalamin Anticancer Derivatives for Targeted Prodrug Delivery via Transcobalamin-Mediated Uptake." *Dalton Trans.* 46, no. 7 (2017): 2159–64. https://doi.org/10.1039/C6DT04443C.

Ruggi, A., and F. Zobi. "Quantum-CORMs: Quantum Dot Sensitized CO Releasing Molecules." *Dalton Trans.* 44, no. 24 (2015): 10928–31. https://doi.org/10.1039/C5DT01681A.
